# ULBERT: a domain-adapted BERT model for bilingual information retrieval from Pakistan's constitution

**DOI:** 10.3389/fdata.2025.1448785

**Published:** 2025-09-22

**Authors:** Qaiser Abbas, Waqas Nawaz, Sadia Niazi, Muhammad Awais

**Affiliations:** ^1^Department of Computer Science, Faculty of Computer and Information Systems, Islamic University of Madinah, Madinah, Saudi Arabia; ^2^Department of Information Systems, Faculty of Computer and Information Systems, Islamic University of Madinah, Madinah, Saudi Arabia; ^3^Department of Psychology, Faculty of Social Sciences, University of Sargodha, Sargodha, Pakistan; ^4^Department of Computer Science, Faculty of Computing and Information Technology, University of Sargodha, Saroghda, Pakistan

**Keywords:** legal information, BERT, constitution, information retrieval, cosine similarity, embedding vectors

## Abstract

**Introduction:**

Navigating legal texts like a national constitution is notoriously difficult due to specialized jargon and complex internal references. For the Constitution of Pakistan, no automated, user-friendly search tool existed to address this challenge. This paper introduces ULBERT, a novel AI-powered information retrieval framework designed to make the constitution accessible to all users, from legal experts to ordinary citizens, in both English and Urdu.

**Methods:**

The system is built around a custom AI model that moves beyond keyword matching to understand the semantic meaning of a user's query. It processes questions in English or Urdu and compares them to the constitutional text, identifying the most relevant passages based on contextual and semantic similarity.

**Results:**

In performance testing, the ULBERT framework proved highly effective. It successfully retrieved the correct constitutional information with an accuracy of 86% for English queries and 73% for Urdu queries.

**Discussion:**

These results demonstrate a significant breakthrough in enhancing the accessibility of foundational legal documents through artificial intelligence. The framework provides an effective and intuitive tool for legal inquiry, empowering a broader audience to understand the Constitution of Pakistan.

## 1 Introduction

The most important law in Pakistan is the Constitution, also called the 1973 Constitution (Shehzaad Nakhoda, 1973). Zulfiqar Ali Bhutto's government wrote it with help from the opposition party, and it was signed into law on 14 August 1973, after being accepted by Parliament on 10 April 1973 (Wynbrandt, 2009). Pakistan's laws, political culture, and political system are based on the rules established by the Constitution. The document addresses the state's physical existence and borders, the fundamental rights of its citizens, constitutional law and order, the national constitutional framework, and the establishment of organizations and the military. The first three chapters outline the roles and duties of the three branches of government. These are a legislature with two houses, an executive branch under the Prime Minister's leadership, and a federal court system with the Supreme Court at its apex. The Constitution's first six articles declare the country a federal parliamentary republic and designate Islam as the state religion. Parts of the Constitution require laws consistent with the Islamic teachings of the Quran and Sunnah. The Constitution of Pakistan is available in print in government offices and online (Shehzaad Nakhoda, 1973). Lawyers, the public, various organizations, the Ministry of Law, and law enforcement agencies typically consult physical copies to address their legal questions. Insufficient knowledge of the Constitution can lead to errors or violations. The search for legal counsel is often expensive and time-consuming, as lawyers must manually search multiple volumes to locate relevant clauses. The identified inefficiency necessitates the development of an information retrieval system based on user queries to search the Constitution of Pakistan.

Information retrieval (IR) is the science of searching for information in a document or file and looking for metadata that describes data and data sets from texts, photos, or sounds (Singhal et al., 2001). The IR process starts when a user sends a query to the system. Queries are straightforward ways to ask for related information. However, unlike traditional database SQL queries, information retrieval results may or may not match the query; therefore, results are usually in order. An IR model selects and ranks the information according to user needs defined in the query. A search engine is software that allows you to look for things on the Internet based on text-based search queries (Erdmann et al., 2022). Search results are usually listed in a row, called search engine result pages (SERPs), which may contain links to web pages, photos, infographics, research papers, etc. Some search results also look through open directories and database files to find information. There are a lot of search engines on the Internet now, and each has its own set of features and ways to provide relevant information, e.g., Archie, Google, AOL, Ask.com, Baidu, Bing, DuckDuckGo, and Yahoo. Similarly, legal IR systems exist to query related information but are limited to certain legal domains, e.g., CourtListener,^1^ FindLaw,^2^ Justia's,^3^ Legal Information Institutes,^4^ Fastcase,^5^ and AI Attorney.^6^ Fastcase, one of the world's largest online law libraries, gives online access to case law, statutes, regulations, constitutions, court rules, and law review articles, making legal research and analysis more efficient and effective. CourtListener is a website for legal research that the non-profit Free Law Project runs. It has court rulings from both the state and federal levels worth billions of dollars. FindLaw for Lawyers is designed to make it easier for everyone to know the law. It has free legal content online, such as case law from courts, summaries of cases, information about government activities, and legal news. Justia's has an enormous and free database of case law, standards, laws, regulatory requirements, and articles regarding federal and state cases. AI Attorney provides paid legal services in Pakistan. The Legal Information Institute (LII) at Cornell Law School gives free online access to most US statutes, making it a valuable resource for legislation, regulation, and legal publications. The current landscape of legal AI includes prominent tools like the AILawyer mobile app.^7^ However, such platforms have notable constraints. AILawyer's claim of covering 150 constitutions, for example, is difficult to substantiate due to a lack of transparency regarding its data sources. Moreover, its commercial nature not only limits access to paying users but is also accompanied by a lack of scientific validation; the tool's technical foundations are discussed only in proprietary blog posts rather than in published, peer-reviewed research. This context reveals a critical need for accessible, transparent, and academically grounded tools, especially for underserved domains. To our knowledge, there is no such legal IR system available for the Constitution of Pakistan.

In this study, we introduce ULBERT (Urdu Legal BERT), a novel domain-adopted BERT model for an offline Information Retrieval (IR) system designed for the bilingual Urdu-English text of the Constitution of Pakistan. While advanced systems like Legal-BERT (Chalkidis et al., 2020) and LexNLP (Bommarito II et al., 2021) exist, their focus on different legal and linguistic frameworks makes direct performance comparisons with ULBERT inappropriate. The fundamental differences in legal terminology, language structure, and judicial concepts necessitate a tailored approach for the Pakistani context. Our methodology is built upon the Bidirectional Encoder Representations from Transformers (BERT) model (Devlin et al., 2018), selected for its state-of-the-art ability to capture deep contextual information, surpassing earlier embeddings like GloVe (Pennington et al., 2014), Word2vec (Mikolov et al., 2013), and ELMo (Peters et al., 2018). The primary contributions of this work are as follows.

- The pioneering application of vector-based IR to the Urdu legal domain, a significantly under-resourced area.
- The fine-tuning of a pre-trained BERT model (Devlin et al., 2018) on a custom, bilingual legal dataset derived from the Constitution of Pakistan.
- We discuss the IR system architecture using our fine-tuned BERT model.
- An empirical analysis evaluating the effectiveness of our proposed system against established baseline methods.
- The development and contribution of a specialized bilingual corpus for the Pakistani legal domain, addressing the critical need for domain-specific training data as highlighted in prior work (Chalkidis et al., 2020).

The remainder of this paper is structured as follows: Section 2 reviews relevant literature, including other legal BERT variants. Section 3 details our proposed methodology, from data acquisition (Section 3.2) and pre-processing (Section 3.3) to model fine-tuning (Sections 3.4–3.8). Section 4.5 presents our experimental results and discussion based on the evaluation metrics from Section 4.4. Finally, Section 5 provides our findings and suggests directions for future research.

## 2 Related works

The information retrieval systems have a long history in terms of using various approaches such as Term Frequency Inverse Document Frequency (TF-IDF) (Leskovec et al., 2020), Probabilistic metrics to rank documents (Dang et al., 2022), Latent Semantic Indexing (LSI) (Rosario, 2000), connection investigation strategies such as PageRank and HITS (Sen and Chaudhary, 2017), etc. These approaches are exact (Boolean) or best (probabilistic and advanced) matching methods. These approaches are good at detecting a few critical components with opaque representations. However, they cannot accurately represent the semantic content of documents in a human-interpretable way, making data analysis difficult (Silva et al., 2019). To overcome the issue of semantic understanding, various word embedding approaches were introduced, such as Word2vec (Mikolov et al., 2013), Glove (Pennington et al., 2014), BERT (Devlin et al., 2018), FastText (Bojanowski et al., 2017), and ELMO (Peters et al., 2018). Among this list, the BERT is a better model that takes advantage of the surrounding text to help computers determine the meaning of confusing words in the text (Getzen et al., 2022). BERT is built on Transformers, a deep learning system in which each result component is connected to each information element, and their respective weightings are substantially influenced by how they are linked. This BERT is already configured to perform two NLP tasks: Masked Language Modeling(MLM) and Next Sentence Prediction (NSP). The Masked Language Model training aims to conceal a word within a sentence and have the product determine which word was concealed, depending on the location of the secret word. The Next Sentence Prediction training aims to get the product to predict whether two given sentences are coherent or not. BERT uses a different input segmentation. BERT splits the input into sub-word units called word pieces. BERT learns its unique word-piece embeddings along with the rest of the model. Because they are frequently merely word fragments, they cannot carry the same semantic information as Word2vec or Glove. Other word embedding language models could only read text from left to right or right to left, not both ways at once, but BERT is unique because it can go in both directions at once due to Transformers. Therefore, the proposed approach uses a pre-trained BERT model (Devlin et al., 2018), available as an open source.

The adaptability of the BERT model has made it a basis in modern machine learning, particularly in hybrid systems. Instead of using BERT alone, many researchers pair its powerful language representations with other algorithms to tackle specialized problems. This approach is common in text classification. In sentiment analysis, for instance, researchers have combined BERT with tools like AdaBoost and Particle Swarm Optimization to parse the nuances of employee reviews (Markovic et al., 2024; Babic et al., 2024). A similar strategy has proven effective in identifying harmful online content, where multi-layered frameworks using classifiers like XGBoost can detect abusive language, cyberbullying, and harassment (Todorovic et al., 2025; Dobrojevic et al., 2024; Mladenovic et al., 2024). While these studies highlight BERT's impressive adaptability, our work ventures into the highly structured and demanding domain of law. Our direction is guided by the pivotal findings of Chalkidis et al. (2020), who demonstrated that fine-tuning pre-trained models on domain-specific text is crucial for achieving the best results. Following this principle, we have focused on applying a custom-tailored BERT model to the under-resourced field of Pakistani constitutional law. In doing so, we introduce a novel application and contribute a valuable new corpus to the natural language processing community.

Several domain-specific, fine-tuned variants of pre-trained BERT have emerged for legal text processing. Legal_BERT (Chalkidis et al., 2020), trained on EU, UK, and US legal corpora, achieved a maximum F-score of 59.2% on the ECHR-CASES dataset with multi-classification. AraLegal-BERT (Al-qurishi et al., 2022), developed for Arabic legal documents, reported precision, recall, and F-score values of approximately 0.89 on the BoG dataset and 0.92 on the SJP dataset, raising questions about the near-identical results. jurBERT (Masala et al., 2021) utilized Romanian legal datasets and evaluated performance using AUC. SM-BERT-CR (Vuong et al., 2023), while building upon similar principles as Chalkidis et al. (2020), employed a different corpus and focused on query-based retrieval of legal cases, achieving a precision of 0.6395, recall of 0.8050, and F-score of 0.6528 on the COLIEE 2020 test set. Notably, SM-BERT-CR also incorporated the TextRank algorithm and GLOVE embeddings (Pennington et al., 2014), differentiating it from the other models (Chalkidis et al., 2020; Al-qurishi et al., 2022; Masala et al., 2021), despite its foundation in fine-tuning a pre-trained BERT model (Devlin et al., 2018).

While several advanced legal IR systems exist, such as Legal-BERT and LexNLP, primarily targeting English and European legal frameworks, direct comparisons with ULBERT are challenging. These systems are trained on datasets and legal systems that differ fundamentally from the Pakistani Constitution and the Urdu language in structure, terminology, and legal concepts. A meaningful comparison would require comparable datasets and evaluation metrics across vastly different linguistic and jurisdictional contexts. ULBERT's primary contribution lies in pioneering the application of vector-based IR to the Urdu legal domain, establishing a foundation for future research in this under-resourced area. Although a comparative evaluation is valuable, the main contribution of this research is its novel focus on a previously unaddressed language and legal system.

In this study, the pre-trained BERT (Devlin et al., 2018) base version was fine-tuned on the legal dataset, which contains the text of the Constitution of Pakistan (CP-Data) as discussed in Section 3.2. The detailed design of our proposed system with the ULBERT model for the legal domain of Urdu is presented next.

## 3 Methodology

### 3.1 System design overview

**Figure 1:**
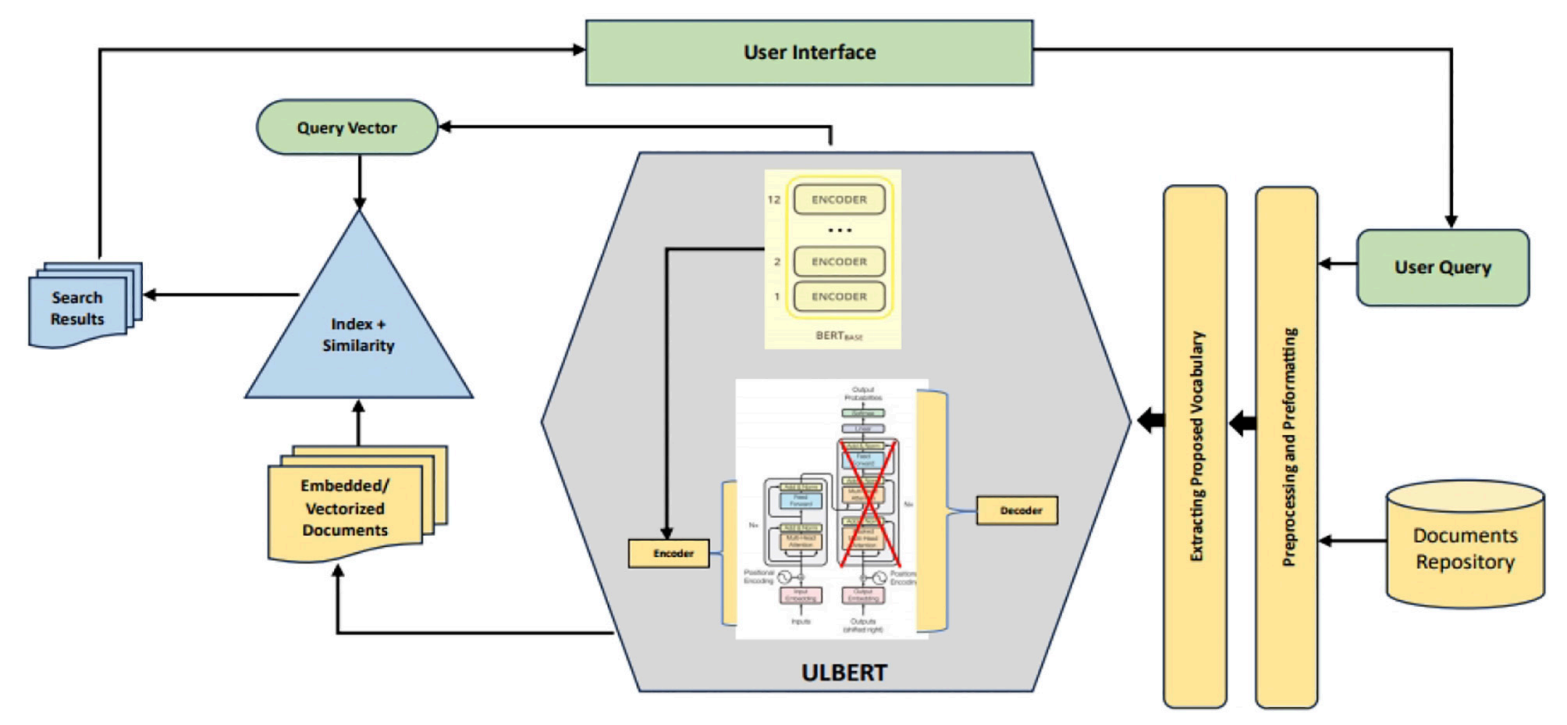
Proposed architecture for IR system using fine-tuned BERT (ULBERT).

The proposed system, ULBERT (Urdu Legal BERT), is designed to be an intelligent search assistant for legal professionals working with the Constitution of Pakistan. Unlike general-purpose search engines like Google, ULBERT focuses on this legal domain, making it a specialized enterprise search solution. This approach centers on fine-tuning the pre-trained base BERT model. The novelty of this work lies not in developing a new architecture, but in demonstrating the successful adaptation of a powerful model to the unique domain of the Pakistani Constitution and its Urdu legal text. Figure 1 provides a visual overview of the system's architecture.

At its core, ULBERT moves beyond traditional keyword-based searches. Instead of simply matching words, it understands the meaning behind the user's query and the legal documents. This is achieved through a vector-based approach. Keyword searches often struggle with complex questions, synonyms, long queries, or when users aren't familiar with the precise legal terminology. ULBERT overcomes these limitations using a cutting-edge language model to create numerical representations (vectors) of the text's meaning. These vectors are then indexed in a high-dimensional space, allowing us to measure the similarity between a user's query vector and the vectors representing each document.

To accomplish its objective, the study leveraged the power of the Bidirectional Encoder Representations from Transformers (BERT) model, specifically utilizing the base-BERT version built upon the Transformer architecture. This model was selected to balance accuracy with computational efficiency, as larger BERT variants, despite offering potentially marginal accuracy gains, would significantly increase computational demands and negatively impact the system's scalability. The underlying Transformer architecture is composed of a series of encoders, where each encoder contains two key components: a multi-head attention layer for focusing on different parts of the input text simultaneously, and a feed-forward neural network. The implemented system employs an architecture of 12 encoder layers, which encompasses a vast network of 110 million parameters. Decoder layers were deliberately excluded from the model, as they are not required for the information retrieval and search tasks central to this research.

The key stages involved in the process are as follows:

- **Data preparation:** The process begins with the careful preparation of the legal document dataset, which involves thorough text cleaning and formatting.
- **Vocabulary building:** Subsequently, the key vocabulary is extracted from this prepared dataset to form a domain-specific lexicon.
- **Fine-tuning BERT:** This vocabulary, along with the dataset, is used to fine-tune a pre-trained base-BERT model, specializing it for Pakistani constitutional law. The base-BERT model automatically generates three types of embeddings (positional, segment, and token) for each word, representing its position, sentence context, and token identity within the input sequence. These embeddings are then processed and refined through the model's series of encoder layers.
- **Index creation:** This fine-tuning process yields a specialized model, termed “ULBERT,” and creates a corresponding dense index of embedding vectors that represent all the documents in the dataset.
- **Query processing:** When a user submits a search query, the ULBERT model transforms the input text into a corresponding high-dimensional query vector.
- **Similarity matching:** The query vector is then compared to the indexed document vectors using the Euclidean distance similarity measure. This calculation determines the semantic proximity between the user's query and each document.
- **Result presentation:** Finally, the documents that are most semantically similar to the query are retrieved and presented to the user as the search results.

The overall design of ULBERT incorporates several advanced techniques, including knowledge distillation, the power of BERT with Transformers, quantization, and pruning. These techniques work together to ensure the system's accuracy and efficiency. The design includes stages of data acquisition with pre-processing. Then, training the ULBERT language model over the CP-Data dataset is performed to obtain embeddings for sentences and words. Input query conversion using those embeddings is the next step. These embeddings are matched using ULBERT, Euclidean Distance, and Cosine Similarity. Finally, the evaluation of the system is done. Details for each of these steps are presented in the following sections.

### 3.2 Data acquisition

The Constitution of Pakistan sets the governing rules for the nation's laws and political system. The opening chapters lay out the structure and duties of the three branches of government: the two-house Legislature (Parliament), the Executive led by the Prime Minister, and the Judiciary, which is overseen by the Supreme Court. The first six provisions of the constitution state that the government is a federal parliamentary republic and Islam is the official religion of the country (Wynbrandt, 2009). Some parts of the Constitution say the legal system must follow the Islamic rules in the Qur'an and the Sunnah. Although the constitution of Pakistan was written in 1973, Republic Day is celebrated every year on March 23, when the first ensemble was released in 1956. Technologically, there have been 26 changes, and only 23 were put into the constitution, and the parliament turned down 3. This study uses the latest version of the Constitution in English and Urdu. First, the data set is filtered to 300 pages for each version in English^8^ and Urdu.^9^ The filter is needed to remove unnecessary structural information from the data set. However, the structure of the Constitution volumes for both the English and Urdu versions is similar. This combined 600-page limit will be increased. Parts, chapters, annexures, and schedules exist in the CP-Data data set. The total size of the CP-Data for English is 341,529 words, and the total size of the CP-Data for Urdu is 366,388, while the individual size of each section is provided in Table 1.

**Table 1:** Statistics of CP-data (dataset).

**Sr. No**.	**Description**	**Size in words**	الفاظکیگنتی	فہرست
1	Part 1, Chapter 1	22,350	۲۴۱۳۸	حصہاول،ابتدائیہ
2	Part 2, Chapter 1–2	13,640	۱۴۵۹۴	حصہدوم،باب۱۔۲
3	Part 3, Chapter 1–3	73,546	۷۹۴۲۹	حصہسوم،بابا۔۳
4	Part 4, Chapter 1–3	12,735	۱۳۸۸۱	حصہچہارم،بابا۔۳
5	Part 5, Chapter 1–3	10,308	۱۱۰۲۹	حصہپنجم،باب۱۔۳
6	Part 6, Chapter 1–3	65,489	۶۹۴۱۸	حصہششم،باب۱۔۳
7	Part 7, Chapter 1–4	35,624	۳۸۱۱۷	حصہہفتم،باب۱۔۴
8	Part 8, Chapter 1–2	11,357	۱۲۲۶۵	حصہہشتم،باب۱۔۲
9	Part 9,10,11	12,353	۱۳۴۶۴	حصہنہم،دہم،یازدہم
10	Part 12, Chapter 1–7	29,367	۳۱۷۱۶	حصہدوازدہم،باب۱۔۷
11	Annex	1,460	۱۵۶۲	ضمیمہ
12	Schedule 1–5	22,379	۲۳۷۲۱	جدول۱۔۵
13	Amendments 1–4	11,683	۱۲۳۸۳	ترامیم۱۔۴
14	Amendments 5–8	13,832	۱۴۹۳۸	ترامیم۵۔۸
15	10th Amendments	578	۶۱۸	ترمیمدہم
16	Amendments 12–15	4,137	۴۳۸۵	ترامیم ۱۲ ۔ ۱۵
17	16th Amendments	689	۷۳۰	ترمیمشانزدہم
	**Total**	**341,529**	۳۶۶۳۸۸	مجموعہ

### 3.3 Preprocessing

An effective pre-processing stage is critical for ensuring good retrieval performance while efficiently managing computational space and time requirements. The primary goal of this stage is to extract essential features and key phrases from the documents within the dataset to improve the semantic relevance of the text. The advent of sophisticated word embedding models, such as Word2vec (Mikolov et al., 2013), Glove (Pennington et al., 2014), Elmo (Peters et al., 2018), and BERT (Devlin et al., 2018), has led to a division of pre-processing into two main categories: traditional techniques and the specialized pre-formatting required by these models. As the proposed approach is based on the BERT architecture, its methodology incorporates a blend of both strategies. The traditional component encompasses standard techniques like tokenization, stop-word removal, and the handling of digits, hyphens, and punctuation. Due to the commonality of these procedures and for the sake of brevity, these steps are not discussed in extensive detail. The focus of this section is therefore placed on the specific pre-formatting required for the ULBERT model.

**Figure 2:**
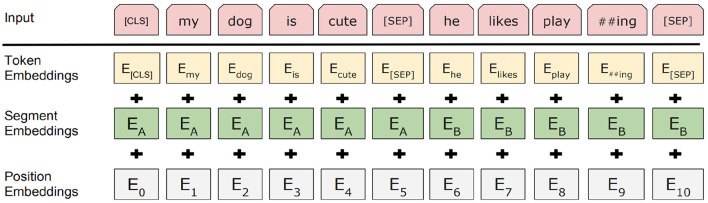
English input embeddings of the ULBERT (image taken from Devlin et al., 2018).

**Figure 3:**
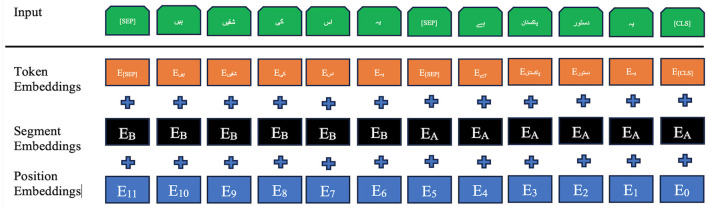
Urdu input embeddings of the ULBERT.

Before fine-tuning the base BERT model on the CP-Data dataset for English and Urdu, the text needs to go through a careful pre-formatting stage. This step generates the positional, segmental, and token embeddings, shown for English in Figure 2 and for Urdu (read right to left) in Figure 3. These embeddings are created through the Masked Language Modeling (MLM) and Next Sentence Prediction (NSP) tasks, both core features of BERT. In this setup, the special [CLS] token marks the start of an input sequence, while [SEP] signals the end of each sentence. Positional embeddings, labeled *E*_0_ through *E*_10_, track where each token appears in the sequence, and segmental embeddings, *E*_*A*_ and *E*_*B*_, identify which sentence a token belongs to. On top of the standard architecture, we add a new document embedding layer right after tokenization. This extra layer, absent from the usual BERT diagrams, is explained in more detail in Section 3.6.

### 3.4 Extracting vocabulary

The next step was to build a domain-specific vocabulary from the legal dataset. Because the original BERT model (Devlin et al., 2018) relies on the WordPiece (WP) tokenizer but that is not available as open source, we opted for the SentencePiece (SP) tokenizer in unigram mode as a practical replacement (Deepa, 2021). SP worked well overall, though it needed a few small tweaks to fit the standard BERT format. A key practical problem is that training SP on the entire dataset at once is very memory hungry and can crash kernels in low-RAM environments like Google Colab. To avoid that, we build the vocabulary iteratively by processing the data in smaller chunks rather than all at once. We also disabled SP's default beginning-of-sentence and end-of-sentence tokens by setting their indices to −1. Finally, the vocabulary size (VOC_SIZE) was chosen in the usual 32,000–128,000 range, and a set number of placeholder tokens (NUM_PLACEHOLDERS) were reserved for future updates.

**Figure 4:**
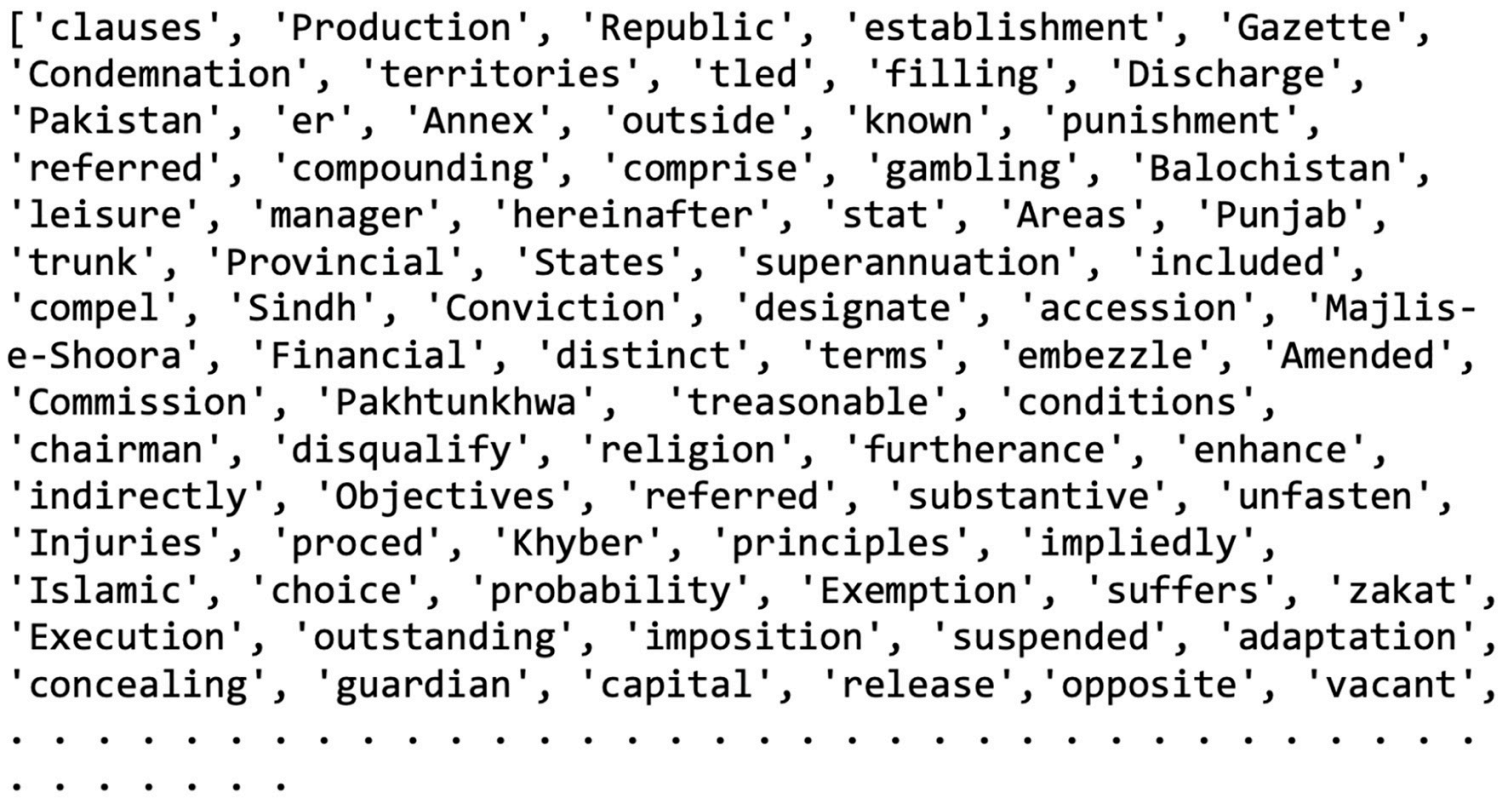
A sample of English vocabulary.

**Figure 5:**
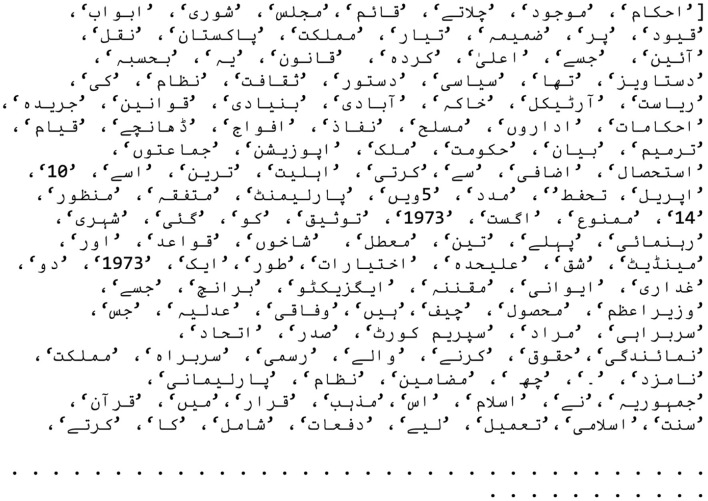
A sample of Urdu vocabulary.

SentencePiece handles tokenization a bit differently than WordPiece. First, it replaces every space with a special marker “_,” so “Hello World.” becomes “Hello_World” before splitting into pieces like [Hello] [_World] [.]. Any word that follows a space gets the “_” prefix; words at the very start of a sentence do not. To make the resulting vocabulary look more like WordPiece, a simple conversion is done: the “_” is removed from SentencePiece tokens and ## is added to sub-word pieces that continue a word. The usual BERT control symbols, [MASK], [SEP], [CLS], [UNK], and so on, are also added along with placeholder tokens. Those placeholders let you introduce new, task-specific tokens (for example, in a legal dataset) by swapping them for real tokens, regenerating the pre-training data, and fine-tuning the model. Finally, the completed vocabulary is saved to a file; examples of the English and Urdu vocabularies are shown in Figures 4, 5.

### 3.5 Generating pre-training data and persistent storage

This section completes the concepts discussed in Sections 3.3, 3.4. The BERT is a bidirectional model that can find the word in a sequence after looking at the preceding and the following context. This search is accomplished using MLM and NSP techniques, mentioned in Section 3.3. In MLM, the missing word is predicted, like fill in the blank for the English/Urdu sentences (Example 0.0.1) provided from the dataset.

The principles set out in this chapter shall be known as the ………… of Policy.

اس باب میں بیان کردہ اصول حکمت عملی کے………. کہلائیں گے

**Figure 6:**
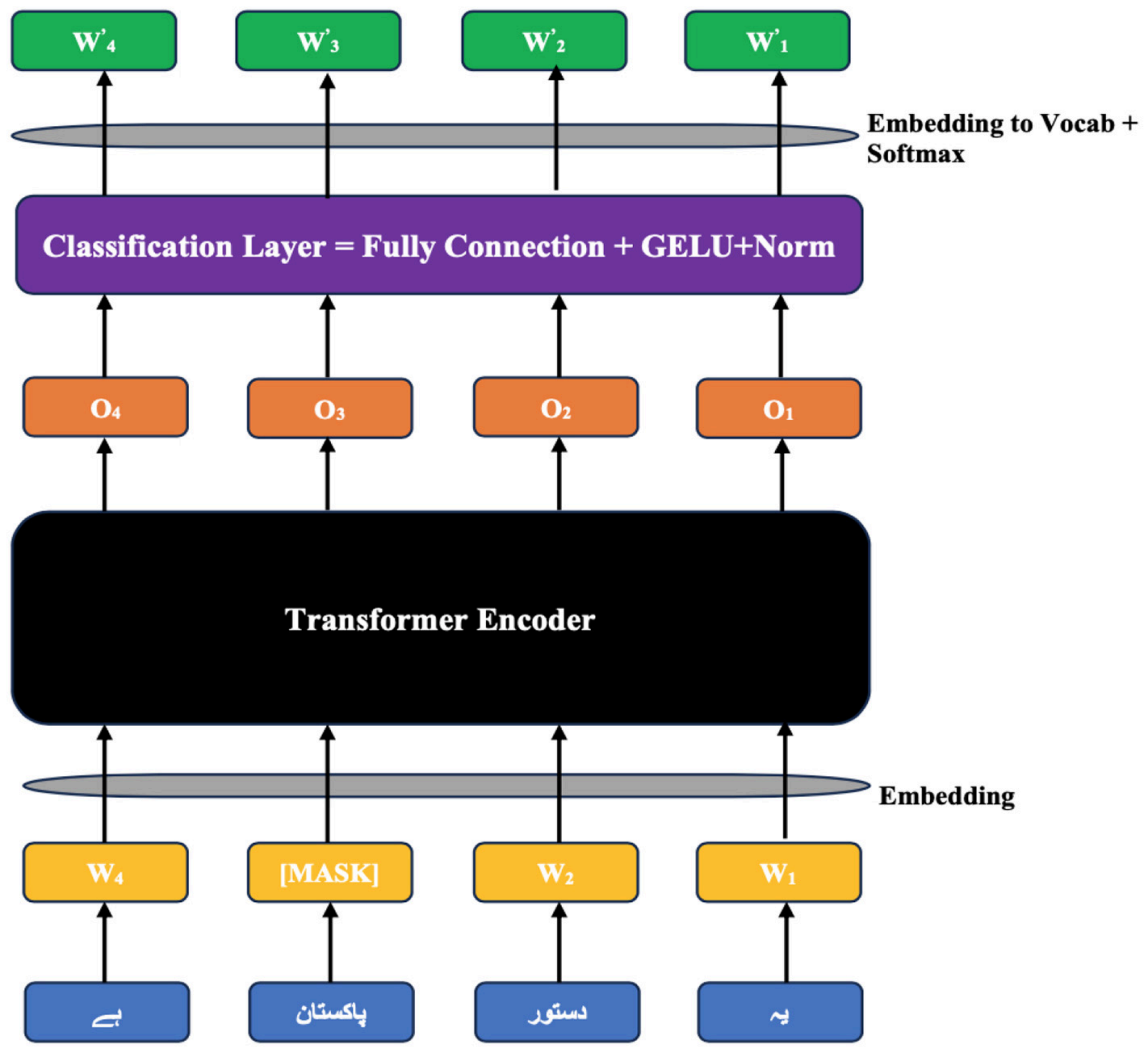
Processing of MLM algorithm.

In MLM, in place of this blank, the hidden word is known as a masked word and labeled with [MASK]. In this pre-training phase, for masking, the MLM algorithm selects 15% of the word positions for prediction from our dataset. For any chosen word position, it performs masking with the [MASK] label 80% of the time, replaces with a random word 10% of the time, and leaves unchanged 10% of the time. The BERT's primary role during training/fine-tuning is to predict the missing words correctly; for this, it uses a classification layer on the encoder output. The vector output of this classification layer is then multiplied by the embedding matrix, which gives us the vocabulary dimensions. This helps in aligning model predictions with the vocabulary, as seen in Figure 6 for Urdu, and the same is true for the case of English vocabulary.

Similarly, in the NSP algorithm, before training, the model selects the pairwise sentences from our dataset. For 50% of pairs, the first and second sentences are according to the actual position of the sentences in the documents and act like a gold standard. While in the other 50% of pairs, the model selects the second sentence randomly. The model's objective is to learn the correct choice of the second sentence using the gold standard. The form of the pre-trained sentences for English and Urdu is depicted in Figures 2 and 3 in Section 3.3. Both the algorithms, MLM and NSP, run together in the base-BERT. However, the MLM is contributing mainly to achieving our objective of the ULBERT enterprise search engine. Technically, the dataset is split into shards. Moreover, the case is unnecessary for Urdu; lowercase is considered valid for English. The model runs with these settings to prepare the pre-training information and takes time, depending on the size of the dataset. The details are presented in Section 3.6.

To store resources persistently, the system uses Google Cloud Storage (GCS). It creates two folders in the bucket—one for the dataset and one for the model, and places the model's vocabulary and configuration files in the model folder. It also sets the BUCKET variable and defines the base-BERT hyperparameters so training can proceed. Once everything is configured, the system uploads the assets to the pre-configured GCS bucket for the next steps, which are described below.

### 3.6 Model training

**Figure 7:**
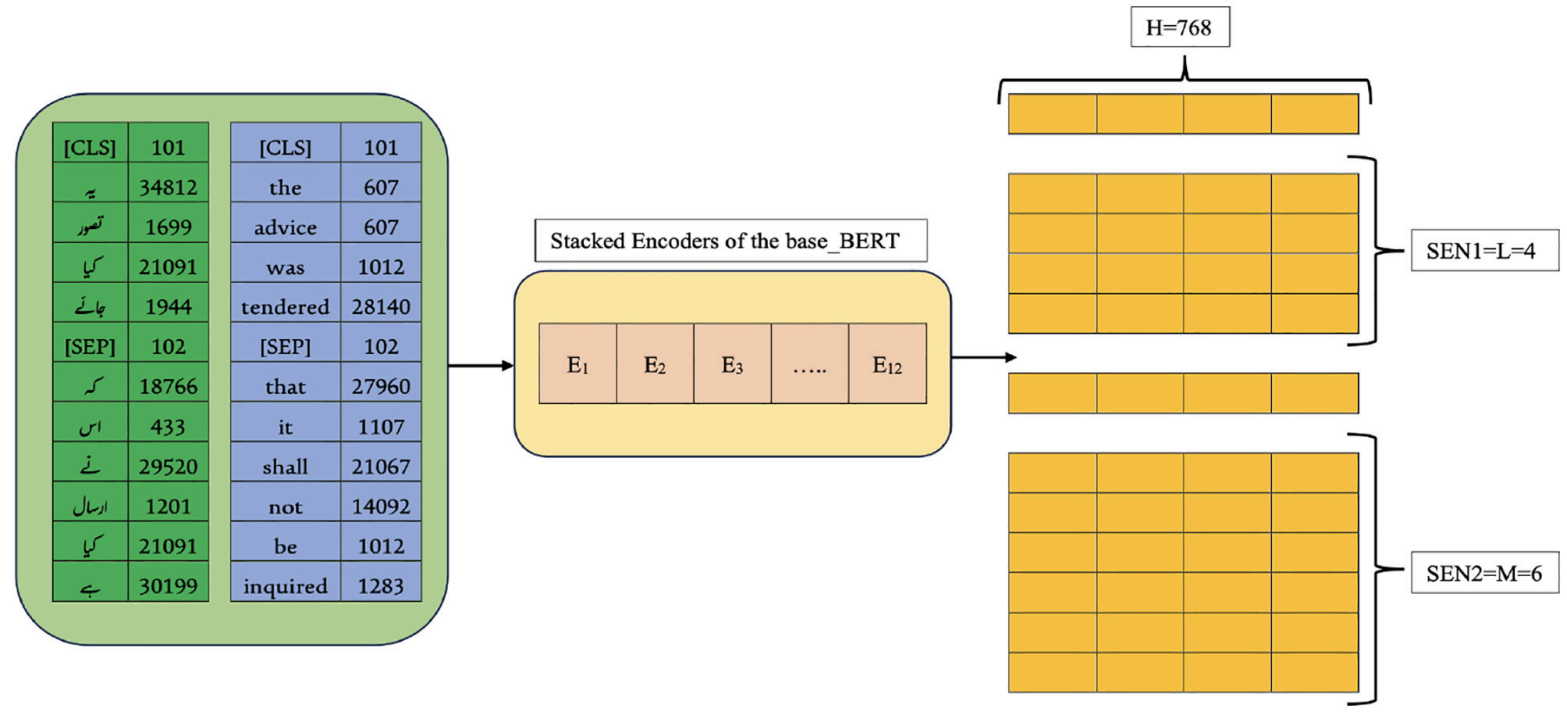
Partial view of ULBERT's training process.

**Figure 8:**
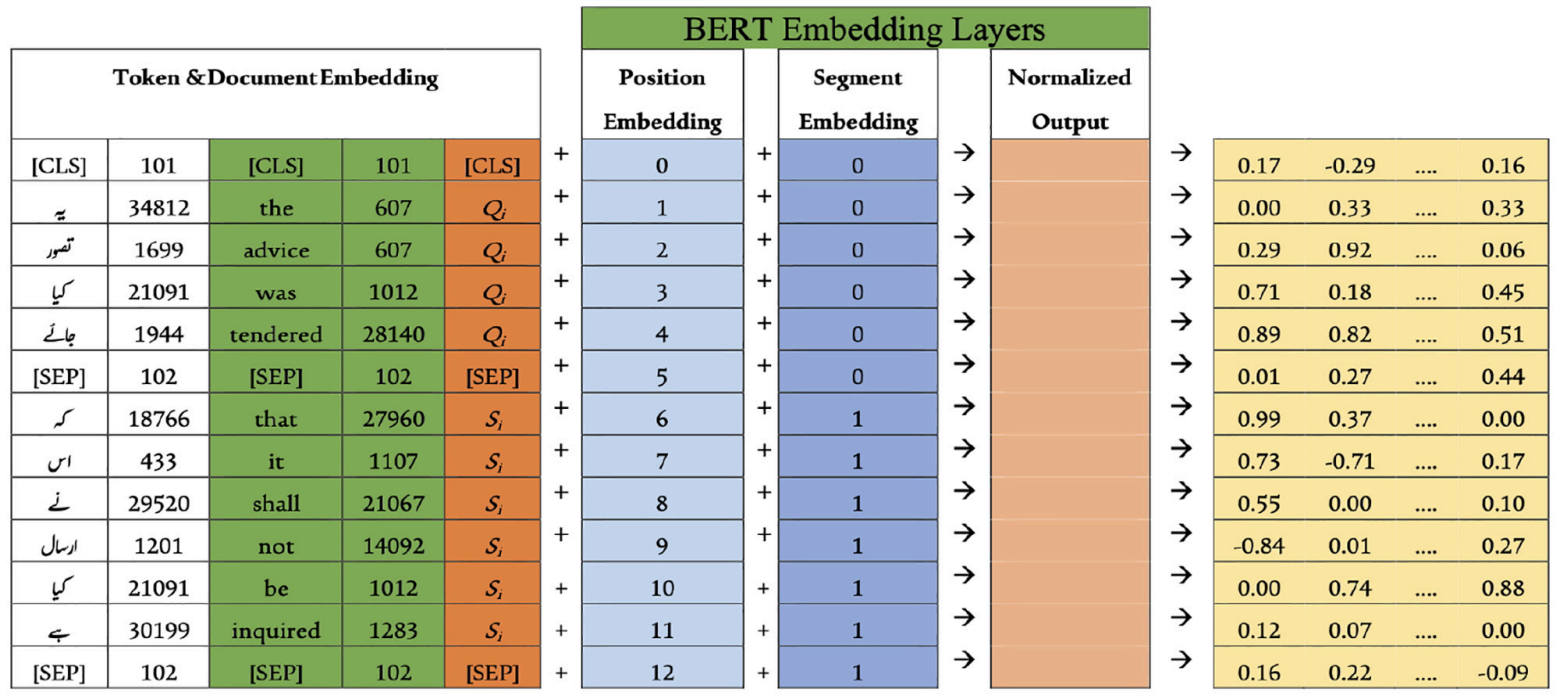
Embedding layer of model.

The fine-tuning process begins with preparing the dataset, as outlined in Section 3.2 and summarized in Table 1. This multilingual dataset is used to fine-tune the base BERT model. Once the data is ready, the model preprocesses the documents, as detailed in Section 3.3. For clarity, both English and Urdu inputs are presented together. The SentencePiece tokenizer is configured to match the WordPiece tokenizer of base BERT, addressing the issues noted in Section 3.4. Using the vocabulary built earlier, the tokenizer generates token IDs for both languages, as shown on the left of Figure 7. To keep the explanation concise, lemma separation during token embedding is not discussed here. After tokenization, the document embedding layer assigns *Q*_*i*_ and *S*_*i*_ values to each token, storing its document ID from the extracted vocabulary. These embeddings are then passed through the segment and position embedding layers, producing a dense vector by summing all three embeddings and applying normalization, as illustrated in Figure 8. Since base BERT accepts sequences up to 512 tokens, shorter inputs are padded with the [PAD] symbol. During training for Next Sentence Prediction (NSP), the model either selects two consecutive sentences (IsNext) from the same document or pairs the first sentence with a random one from the dataset (NotNext). For Masked Language Modeling (MLM), sentences are processed individually to generate the necessary vectors.

The first step in fine-tuning/training is to prepare dataset, discussed in Section 3.2 and Table 1, for our model. This multilingual dataset is used to fine-tune the base-BERT model. The model then preprocesses the documents available in the dataset. The details can be seen in Section 3.3. For brevity, a combined view of English and Urdu input is presented to the model. It configures the SentencePiece tokenizer with the exact specification as the WordPiece tokenizer of base BERT. This is done due to its issues (discussed in Section 3.4). The SentencePiece tokenizer generates the token IDs after looking into the vocabulary created already in Section 3.4, and a view can be seen on the left of Figure 7 for English and Urdu, as well. For clarity, the discussion on separating lemmas during token embedding is left out. Once the tokenization layer is complete, the process moves to the document embedding layer, which assigns each token its corresponding *Q*_*i*_ and *S*_*i*_ values. This layer also keeps track of the document ID linked to every token in the vocabulary, as described earlier in Section 3.4. Afterwards, the vectors of two sentences are passed toward the segment and position embedding layer to obtain a dense vector at the end. This dense vector is achieved by summing these three layers of embeddings of the model depicted in Figure 8, and then normalization is applied to the sums. The maximum length of the input sequence for the base-BERT is 512 tokens, so, by default, the remaining unused places of a short input sequence would be padded with the [PAD] symbol. During training, for NSP, the model selects iteratively two sentences A and B sequentially from the document, like a bigram model, and builds vectors by considering the approach of IsNext() for the whole document. Similarly, the model selects the first sentence A from the document and then any random sentence B from the whole dataset to build the vectors for NotNext(). This IsNext() and NotNext() are defined next. For MLM, the model takes sentences from each document individually and generates the vectors accordingly.

The input sequence to the three embedding layers of the model can be a single sentence or a pair of sentences. For simplicity, the detailed version of NSP is presented, excluding MLM as a component of the model. Both the components are discussed already in Section 3.5. Each dense vector obtained for each input token depicted at the end of Figure 8 is passed to the attention head part. The attention head finds the relationship between tokens of a sentence and outputs to the feed-forward neural network. Here, the size of each dense vector is multiplied by the last 4 layers, producing a hidden matrix H of size 768 × 4 as displayed on the right side of Figure 7, where L and M are the respective sizes of the sentences: SEN1 and SEN2.

**Figure 9:**
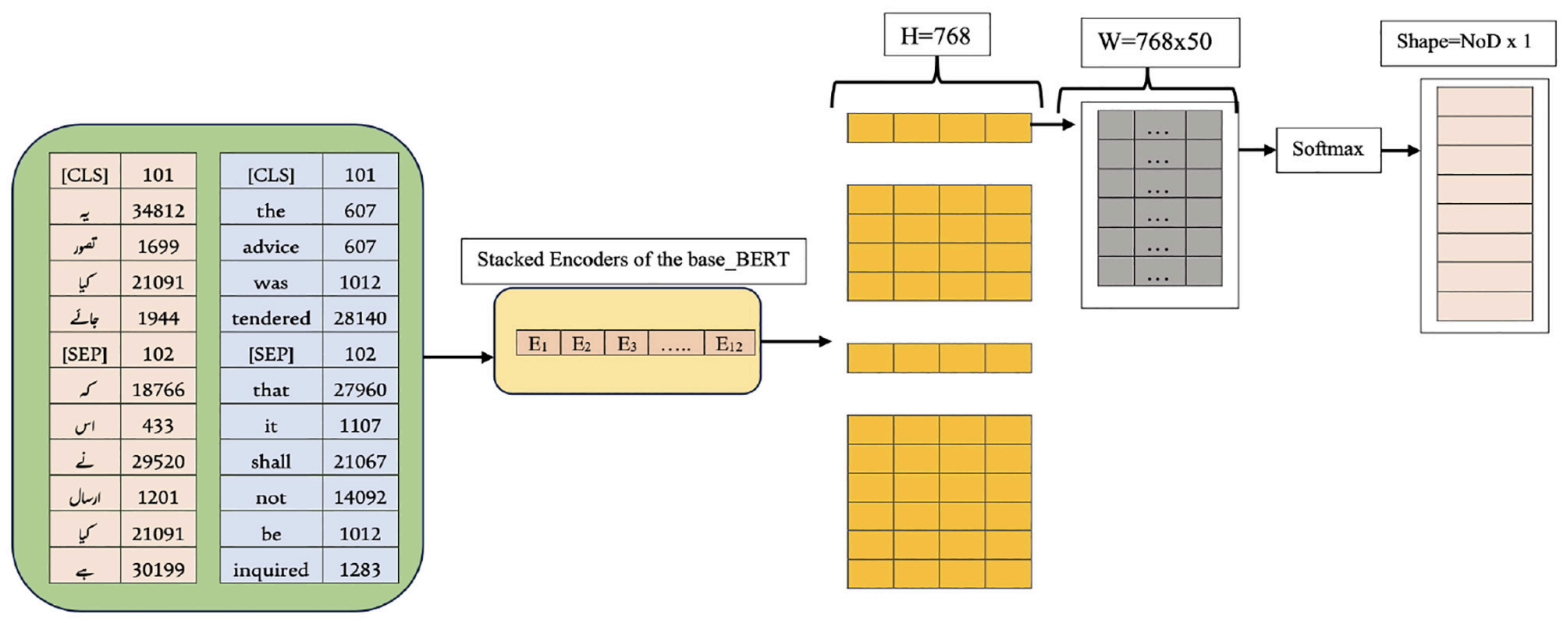
Fine-tuning of our model.

For document retrieval, the model adds a new weight layer W of size H × NoD, where H = 768 is the hidden dimension and NoD (number of documents) equals 50, as shown in Figure 9. A separate W matrix is computed for each row from the previous step, and those weights are optimized with logistic regression using a softmax and cross-entropy loss; L1 regularization is applied to prune parameters. Fine-tuning is handled differently by language: the English portion of the CP-Dataset is fine-tuned from the pre-trained model, while the Urdu portion is trained from scratch because baseBERT lacks a pre-trained Urdu vocabulary. Both language parts use masked language modeling (MLM) with a 15% masking rate that mixes masked, random, and unchanged tokens during training. For example, in a 200-word sequence, 30 tokens (15%) are selected for masking, of which 10% (3 tokens) are replaced with random words and another 10% (3 tokens) are left unchanged. For next-sentence prediction (NSP), each example pairs sentence A with sentence B: 50% of the time, B actually follows A (IsNext), and 50% of the time, B is drawn randomly from the dataset (NotNext); the model casts this as a binary decision using a sigmoid instead of a softmax.

The training ensures the parameters are set consistently with the execution flow. It is followed by the construction of the estimator and the input characteristics. Afterward, the model is executed by calling the train function, i.e., *estimator.train(p)* where p refers to parameters input_fn = train_input_fn, and max_steps = TRAIN_STEPS. The model is trained with the default parameters for 1 million steps, and it took 6~0 hours of run time for English and 6~7 h for Urdu. During this process, the kernel restarted for some reason, but training continued from the latest checkpoint. Our ULBERT customized model has a built-in neural network. After training, the embeddings are produced in the textual content of legal documents. It does not force us to create a custom tokenizer, as the base BERT transformers used in ULBERT include their techniques. It is straightforward and accessible to the best version that produces a set-length vector for each token in the document. Finally, this process yields a trained model stored on a cloud using the bilingual dataset.

### 3.7 Feature extraction for search queries

The proposed search strategy uses the fine-tuned BERT (as discussed in Section 3.6) as a feature extractor for the search queries. For technical configuration, the key steps involved are discussed as follows.

- **Loading fine-tuned and trained model:** This loads our fine-tuned/trained BERT-based model into the memory using simple Python statements. The original base-BERT has the pre-trained vocabulary for English already, but no such pre-trained vocabulary is available for Urdu except (Malik et al., 2023), which is for the classification of abusive/threatening content and is totally different from our legal content. Therefore, the base-BERT (Devlin et al., 2018) is trained for the legal domain completely from scratch. It is to avoid the latency introduced and potential performance issues associated with a client-server architecture. The BERT-base model learns directly from raw text through self-supervision, using masked language modeling (MLM) and next-sentence prediction (NSP). By contrast, supervised models usually rely on labeled examples and can perform well on small, domain-specific corpora when those labels are available. MLM and NSP let BERT extract deep contextual and semantic representations automatically, without human annotations. As Kotei and Thirunavukarasu (2023) note, this self-supervised training helps the model generalize to downstream tasks even when labeled data are scarce. Because the English BERT was trained on a large scale, additional labeled data are often unnecessary. For the Urdu legal dataset, however, fine-tuning ULBERT with labeled examples can improve results. Still, since the project aims to move beyond this single, small dataset, the team has left extensive labeling for future work, favoring the unsupervised approach for now because it is cheaper, easier to scale, and tends to generalize better while reducing dependence on manual annotations. The ULBERT fine-tuned and trained model for English and Urdu, respectively, is discussed in Section 3.6 along with a detailed diagram in Figure 9. Both models are loaded for use from their persistent storage, separately for English and Urdu. This technical configuration step is achieved in a Python environment using simple statements like wget, unzip, pip, etc.
- **Optimizing the inference graph of model:** This is the step to configure the optimization inference graph of the BERT model. First, investigate the suitability of the vector representation size, layers, and input sequence. After this, the model performs or infers calculations optimally and efficiently. The name “optimize_graph” of the library in the original base-BERT is a little misleading. However, the inference graph is configured to obtain a fixed representation of vectors to improve efficiency. This applies a type of pooling named “REDUCE_MEAN,” which averages the vectors for all tokens within the collection. Similarly, this graph model's “SEQ_LEN” variable can be reduced to a smaller value to boost the model's efficiency.
- **Query feature extraction:** Before the fine-tuned/trained ULBERT model, the search query goes through the same steps discussed in Section 3.3. First, the query is passed through the tokenizer to break it into tokens. Additional tokens like [CLS] and [SEP] are added at the query's start and end. Then, the tokens in the query are transformed into vocabulary IDs based on the existing vocabulary obtained in Section 3.4. Then, passing through the segment and position layers, a high-dimensional vector for each query token is generated, as seen in Figures 2, 3 for English and Urdu. The only difference is that the query has a single sentence in our work. The [CLS] token contains the complete representation of the query in base-BERT and acts as a feature vector for the entire query. This same method is applied to generate the document vectors for our CP-Dataset. Afterward, similarity measures like cosine similarity and Euclidean distance are used to find the similarity score. The documents are ranked according to this score, and the top-ranked documents are returned.

### 3.8 Building an information retrieval system

The knowledge base of our dataset contains 600 pages of text samples, and our system needs fast, fact-based answers to users' queries. To find those answers, our approach runs a nearest-neighbor search in a vector space. There are several ways to measure which items are “closest” in that space, but the proposed approach uses Euclidean distance. The proposed text-based information-retrieval system follows the steps below.

1. Vectorizing all document samples from the knowledge base that gives S.
2. Vectorizing the question gives Q.
3. Calculate the Euclidean distance D between Q and S.
4. Sorting D in ascending order- providing indices of most similar samples.
5. Return labels for the samples stated from the information base.

It creates a placeholder for Q and S and then define the Euclidean distance computation. After that, it get the most similar indices. It has a retrieval algorithm using a bit of mathematics as follows. For a pair of vectors p and q, the Euclidean distance is defined in Equation 1 as follows.


(1) $$\begin{array}{l} d\left(p,q\right)=d\left(q,p\right)\\ \;=\sqrt{{\left(q_{1}-p_{1}\right)}^{2}+{\left(q_{1}-p_{1}\right)}^{2}+\cdots +{\left(q_{n}-p_{n}\right)}^{2}}\\ \;=\sqrt{\overset{n}{\underset{i=1}{\sum }}{\left(q_{i}-p_{i}\right)}^{2}}. \end{array}$$

Since p and q are vectors, it can be rewritten as in Equation 2.


(2) $$\begin{array}{r} d{\left(p,q\right)}^{2}=\langle p-q,p-q\rangle \\ =\langle pp\rangle -2\langle pq\rangle +\langle qq\rangle \\ =2*\left(1-\langle pq\rangle \right). \end{array}$$

Here, 〈… 〉 denotes the inner product. In the above formula, pp and qq are squared L2 norms of the vectors shown in Equation 2. These two vectors are L2 standardized in this event, pp = qq = 1. That gives a fascinating connection between internal items and Euclidean distance.

L2 normalization can sometimes remove valuable information about a vector's values, which is often not ideal. To address this, when the knowledge base remains unchanged, the squared vector norm also stays the same. Rather than recalculating it each time, the process computes it once and reuses the result, speeding up distance calculations. This approach works with any vectorizer model, not just the proposed ULBERT, and delivers strong performance in nearest neighbor searches, handling dozens of requests per second on a dual-core Colab CPU.

This concludes the design of our legal ULBERT IR system with a feature extractor. The process of IR is elaborated through an example (see Appendix 6.1). This version is built with the capabilities for in-depth classification and retrieval tasks. Their performance can be further advanced through first-class tuning (see Section 4). The described approach to text feature extraction offers a strong unsupervised baseline for downstream NLP solutions.

## 4 Experiments

In this section, we discuss the experimental details of our proposed IR system, i.e., ULBERT.

### 4.1 Environmental setup

Deep Neural Network (DNN) has recently gained popularity due to better performance and prediction results. Therefore, it is opted to use an existing pre-trained deep learning model known as BERT (Bidirectional Encoder Representations from Transformers) by Devlin et al. (2018) for our experiments. Free GPUs such as Gradient,^10^ Google COLAB,^11^ Kaggle,^12^ etc., can be used to execute our research work, but our approach uses the Google Cloud Platform (GCP) account and Google Cloud Storage (GCS) bucket for persistent storage of training data and model. The source code of the research work is written using Python and Bash commands to run in the COLAB Jupyter environment. It is always better to use the COLAB Jupyter environment when the dataset is significant; however, all our work tasks are performed on a standard machine, except for the training part. This is not without issues that can be resolved through knowledge distillation, quantization, pruning, etc. (Makuvaza et al., 2021).

**Figure 10:**
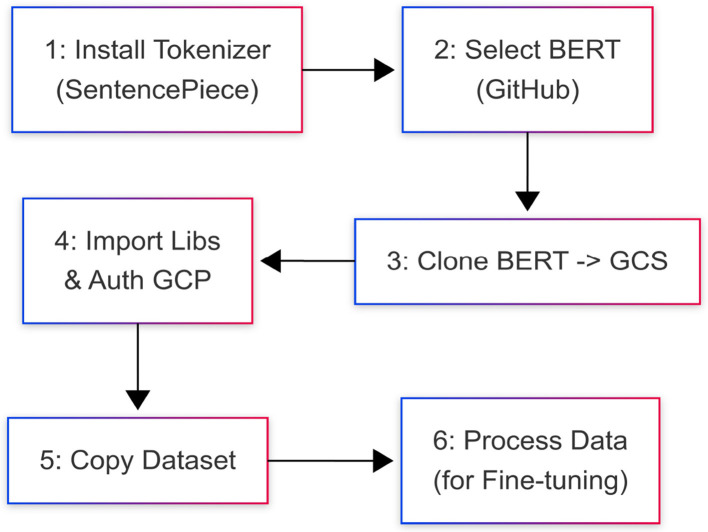
Process of environmental setup.

The following steps are defined to set up the experimental environment, as illustrated in Figure 10.

1. **Install tokenizer:** Use the bash command !pip install SentencePiece to install the SentencePiece tokenizer package. This package is suitable for neural network-based text generation, where the vocabulary size is determined before model training.
2. **Select and clone BERT model:** Choose an open-source BERT model available in a GitHub repository.
3. **Store BERT model:** Clone the selected BERT model repository into a Google Cloud Storage (GCS) bucket using the !git clone [repository_url] bash command. (Remember to replace [repository_url] with the actual URL).
4. **Import libraries and authorize:** Import necessary Python packages such as os, sys, json, nltk, random, logging, TensorFlow, and others as required. Also, complete the necessary authorization steps for accessing Google Cloud Platform (GCP).
5. **Prepare dataset copy:** Copy the dataset to a separate file location for subsequent access during the experiment.
6. **Data processing for fine-tuning:** Acquire the data, perform necessary preprocessing steps, extract the vocabulary, and generate data containing the extracted features. This prepared data will be used for fine-tuning the base BERT model, following the procedures outlined in the methodology section.

### 4.2 Benchmarks for comparison

The proposed system is evaluated against the standard BERT model and other models such as Legal-BERT (Chalkidis et al., 2020), AraLegal-BERT (Al-qurishi et al., 2022), and SM-BERT-CR (Vuong et al., 2023). Though a direct quantitative comparison with other systems like Legal-BERT and LexNLP was impractical due to differences in linguistic, jurisdictional, and data availability, it's important to highlight their distinct focuses. Legal-BERT excels in the broad domain of U.S. legal documents in English, whereas ULBERT is laser-focused on the Urdu language and the specific nuances of the Pakistani Constitution. LexNLP, primarily an NLP toolkit, targets tasks like contract analysis, in contrast to ULBERT's constitutional document retrieval. Our choice to fine-tune a base BERT model, rather than starting from scratch, was driven by the need to leverage pre-existing linguistic knowledge and adapt it efficiently to the unique characteristics of Urdu legal language and the relatively smaller size of our specialized corpus compared to the vast datasets used to train general-purpose English models.

### 4.3 Data set for training and testing

Our proposed model uses the Constitution of Pakistan, discussed in Section 3. Its file size is 45.7 MB. Our data set initially does not have labels. After performing the primary preprocessing, which includes tokenization, stopping, normalization, stemming, etc., a secondary preprocessing is performed. It includes inserting [SEP], [CLS], tokenIDs, DocIDs, maskIDs, segmentIDs, and position embeddings. As discussed in the previous section, our dataset is converted it into vector forms (embeddings). At the same time, each document is labeled with the document ID, as shown in Table 2.

**Table 2:** Data set of constitution of Pakistan (training data).

**Document**	**ID**
Preface	D1
Part I: Introductory [Articles 1–6]	D2
Part II: Fundamental Rights and Principles of Policy [Articles 7–40]	D3
Chapter 1: Fundamental Rights [Articles 8–28]	D4
Chapter 2: Principles of Policy [Articles 29–40]	D5
Part III: The Federation of Pakistan [Articles 41–100]	D6
Chapter 1: The President [Articles 41–49]	D7
Chapter 2: Majlis-e-Shoora (Parliament) [Articles 50–89]	D8
Chapter 3: The Federal Government [Articles 90–100]	D9
Part IV: Provinces [Articles 101–140A]	D10
Chapter 1: The Governors [Articles 101–105]	D11
Chapter 2: Provincial Assemblies [Articles 106–128]	D12
Chapter 3: The Provincial Governments [Articles 129–140A]	D13
Part V: Relations between the Federation and the Provinces [Articles 141–159]	D14
Chapter 1: Distribution of Legislative Powers [Articles 141–144]	D15
Chapter 2: Administrative Relations between the Federation and Provinces [Articles 145–152]	D16
Chapter 3: Special Provisions [Articles 153–159]	D17
Part VI: Finance, Property, Contracts, and Suits [Articles 160-174]	D18
Chapter 1: Finance [Articles 160–165A]	D19
Chapter 2: Borrowing and Audit [Articles 166–171]	D20
Chapter 3: Property, Contracts, Liabilities and Suits [Articles 172–174]	D21
Part VII: The Judicature [Articles 175-212]	D22
Chapter 1: The Courts [Articles 175-175A]	D23
Chapter 2: The Supreme Court of Pakistan [Articles 176-191]	D24
Chapter 3: The High Court's [Articles 192–203]	D25
Chapter 3A: Federal Shariat Court [Articles 203A–203J]	D26
Chapter 4: General Provisions Relating to the Judicature [Articles 204–212]	D27
Part VIII: Elections [Articles 213–226]	D28
Chapter 1: Chief Election Commissioner and Elections Commissions [Articles 213–221]	D29
Chapter 2: Electoral Laws and Conduct of Elections [Articles 222–226]	D30
Part IX: Islamic Provisions [Articles 227–231]	D31
Part X: Emergency Provisions [Articles 232–237]	D32
Part XI: Amendment of the Constitution [Articles 238–239]	D33
Part XII: Miscellaneous [Articles 240–280]	D34
Chapter 1: Services [Articles 240–242]	D35
Chapter 2: Armed Forces [Articles 243–245]	D36
Chapter 3: Tribal Areas [Articles 246–247]	D37
Chapter 4: General [Articles 248–259]	D38
Chapter 5: Interpretation [Articles 260–264]	D39
Chapter 6: Title, Commencement, and Repeal [Articles 265–266]	D40
Chapter 7: Transitional [Articles 267–280]	D41
Annex	D42
The Objectives Resolution	D43
Schedules	D44
First schedule: Laws exempted from the operation of Articles 8(1) and (2)	D45
Second schedule: Election of President	D46
Third Schedule: Oaths of Office	D47
Fourth Schedule: Legislative Lists	D48
Fifth Schedule: Remuneration and Terms and Conditions of Service of Judges	D49
Amendments: 1974, 1975, 1976, 1977, 1985, 1987, 1991, 1997, 1999, 2003, 2010, 2011, etc.	D50

To handle 100 queries in the test data, the ULBERT first converts them to vector forms. Then, it measures the similarity between the query vector and the vectors of the trained documents. Finally, it gets the results in documents ranked in order of cosine similarity. The sample of the query test-data is shown in Table 3.

**Table 3:** Queries test data.

**No**.	**Question**	سوال
1	What are the essential rights?	بنیادیحقوقکیاہیں؟
2	The composition of the Parliament of Pakistan under the charter of the Islamic Republic of Pakistan.	اسلامیجمہوریہپاکستانکےآئینکےتحتپارلیمنٹآفپاکستانکیتشکیل۔
3	What are the safeguards for residents regarding Preventive Detention?	احتیاطیحراستکےحوالےسےشہریوںکےلیےکیاحفاظتیاقداماتہیں؟
4	The powers and role of the president of Pakistan under the constitution of the Islamic Republic of Pakistan, 1993, 1994, 1997.	آئیناسلامیجمہوریہپاکستان۱۹۹۳،۱۹۹۴،۱۹۹۷کےتحتصدرپاکستانکےاختیاراتاورکردار۔
5	The powers and jurisdiction of the High Courts under Article 199 of the Constitution of the Islamic Republic of Pakistan.	اسلامیجمہوریہپاکستانکےآئینکےآرٹیکل 199 کے تحت ہائی کورٹس کے اختیارات اور دائرہ اختیار۔
6	Islamic provisions below the 1973 charter.	۱۹۷۳کےآئینکےتحتاسلامیدفعات۔
7	The system of modification of the charter of the Islamic Republic of Pakistan.	اسلامیجمہوریہپاکستانکےآئینمیںترمیمکانظام۔
8	The powers and jurisdiction of the Federal Shariat Court docket under the charter of the Islamic Republic of Pakistan.	اسلامیجمہوریہپاکستانکےآئینکےتحتوفاقیشرعیعدالتکےاختیاراتاوردائرہاختیار۔
9	Islamic Ideology Council and Election Commission of Pakistan.	اسلامینظریاتیکونسلاورالیکشنکمیشنآفپاکستان۔
10	The ideas of policy are laid down within the charter of the Islamic Republic of Pakistan.	اسلامیجمہوریہپاکستانکےآئینمیںپالیسیکےاصولوضعکیےگئےہیں۔
11	The prime minister of the Islamic Republic of Pakistan below the existing charter 1995, 1997.	اسلامیجمہوریہپاکستانکےموجودہآئین۱۹۹۵،۱۹۹۷کےتحتوزیراعظم۔
12	The family members between Federation and Provinces under the charter of 1973.	۱۹۷۳کےآئینکےتحتوفاقاورصوبوںکےدرمیانتعلقات۔
13	The powers and jurisdiction of the Supreme Court of Pakistan.	سپریمکورٹآفپاکستانکےاختیاراتاوردائرہاختیار۔
14	The various points of emergencies and in whom the power is vested below the constitution.	ہنگامیحالاتکےمختلفپہلواورآئینکےتحتکسمیںاختیاردیاگیاہے۔
15	What are the functions of the superb Judicial Council?	سپریمجوڈیشلکونسلکےکیافرائضہیں؟
…	…	…
40	Diverse modes of Talaaq in Islamic law.	اسلامی قانون میں طلاق کے مختلف طریقے
41	What is the obstacle in inheritance?	وراثتمیںکیارکاوٹہے؟
42	Define the time non-public global regulation.	نجی بین الاقوامی قانون کی تعریف کریں۔
43	In which will Pakistani courts exercise jurisdiction in matrimonial subjects?	پاکستانیعدالتیںازدواجیمعاملاتمیںکسحدتکدائرہاختیاراستعمالکرسکتیہیں؟
44	Diverse methods of pacific or amicable settlements of international disputes below the U. N. Constitution.	اقواممتحدہکےآئینکےتحتبینالاقوامیتنازعاتکےپرامنیادوستانہحلکےمختلفطریقے
…	…	…
100	…	…

In queries from 1 to 100, numerous terms are capitalized and converted to lowercase. The queries contain English contractions, as in No. 44 in the Table. The ULBERT IR system expands these contractions and performs other miscellaneous tasks during the preprocessing.

### 4.4 Evaluation measures

The measures and metrics used for the ULBERT evaluation are discussed below.

#### 4.4.1 Cosine similarity

Cosine similarity is a helpful metric for determining how similar two documents are likely to be in terms of topic content or subject matter, regardless of their length (Chen et al., 2021). One advantage of cosine similarity is its low complexity, especially for sparse vectors. Given vectors of attributes A and B, the cosine similarity, *cos*(θ), is represented as the dot product and magnitude as displayed in Equation 3.


(3) $$\begin{array}{r} SimCos\left(A,B\right):=cos\left(\theta \right)\\ =\frac{A.B}{\left|A||B\right|} \end{array}$$

$$\begin{array}{l} =\frac{\overset{n}{\underset{i=1}{\sum }}\left(A_{i}B_{i}\right)}{\sqrt{\overset{n}{\underset{i=1}{\sum }}{\left(A_{i}\right)}^{2}}\sqrt{\overset{n}{\underset{i=1}{\sum }}{\left(B_{i}\right)}^{2}}}. \end{array}$$

Here, *A*_*i*_ and *B*_*i*_ are vectors A and B. The ensuing similarity scale runs from –1 to 1, with 0 representing orthogonality or no correlation. The positive values up to 1 indicate similarity, and the negative values up to –1 represent dissimilarity.

This measure is language-independent and useful in cases involving frequency rather than absolute values. In the texts of languages like English and Urdu, the words, phrases, and sentences have frequencies. Due to this, the vocabulary of documents is converted into vectors by using various methods available in information retrieval. A query can also be represented in the form of a vector. The cosine similarity of two vectors is cosθ, which can be measured with θ. When θ = 0, sentences A and B overlap; if θ = 90, then sentences A and B are dissimilar. If the degree of θ is between 0 and 90, it represents the degree of similarity between the two sentences.

Cosine similarity may normalize the document length when comparing them because the time frequencies can be suitable. Similarly, the time-cosine distance is usually used to complement cosine similarity in positive space. The cosine distance, then again, is not an appropriate distance metric since it needs the triangle inequality property. The cosine distance is half of the squared Euclidean distance, and the squared Euclidean distance does not meet the triangle inequality. It is essential to convert it into an angular or an Euclidean distance to fix the triangle inequality property while preserving the exact ordering. Similarities between pairs of features are considered using a soft cosine between two vectors. The soft cosine measure proposes considering the similarity of features in the vector space model, which helps to generalize the concept of cosine (and soft cosine) and the idea of (soft) similarity. In natural language processing (NLP), for example, the similarity of features is obvious.

Given two N-dimensional vectors, a and b, the soft cosine similarity is calculated as in Equation 4.


(4) $$\begin{array}{l} Sim_{soft}\left(a,b\right)=\frac{\overset{N}{\underset{i,j}{\sum }}\left(s_{ij}a_{i}b_{j}\right)}{\sqrt{\overset{N}{\underset{i,j}{\sum }}\left(s_{ij}a_{i}a_{j}\right)}\sqrt{\overset{N}{\underset{i,j}{\sum }}\left(s_{ij}b_{i}b_{j}\right)}}. \end{array}$$

where *s*_*ij*_ = *similarity*(*feature*_*i*_, *feature*_*j*_). Suppose there is no similarity between features (*s*_*ii*_ = 1, *s*_*ij*_ = 0 for *i*≠*j*). In that case, the given equation is equivalent to the conventional formula of cosine similarity in Equation 3. The time complexity of this measure is quadratic, making it applicable to real-world tasks. Furthermore, the complexity can be reduced to a square root.

The most notable feature of cosine similarity is that it represents a relative rather than an absolute comparison of the distinct vector dimensions. As a result, the measure is best suited to data in which frequency is more relevant than absolute values, such as phrase frequency in documents. In short, the cosine distance can be expressed in terms of the Euclidean distance as in Equation 4.


(5) $$\begin{array}{l} Dis_{cos}\left(A,B\right)=\frac{|A-B{|}^{2}}{2}\\ \;when\;|A{|}^{2}=|B{|}^{2}=1. \end{array}$$

Equation 5 is derived from the Euclidean distance to measure the nearest-neighbor retrieval to evaluate the ULBERT in Equation 2. The Euclidean Distance measure is used due to its computational efficiency during similarity matching. This contributes to fast query response times. The effectiveness of this measure is highlighted with an example in Appendix 6.2.

#### 4.4.2 Precision and recall

Precision and recall measure how well a system groups things or finds information. As shown in Equation 6, precision is the number of relevant instances out of all retrieved instances. The recall or sensitivity is the percentage of relevant cases, as shown in Equation 7.


(6) $$\begin{array}{l} precision=\frac{tp}{tp+fp}\\ \;=\frac{retrieved\;and\;relevant\;documents}{all\;retrieved\;documents}. \end{array}$$

(7) $$\begin{array}{l} recall=\frac{tp}{tp+fn}\\ \;=\frac{retrieved\;and\;relevant\;documents}{all\;relevant\;documents}. \end{array}$$

Where *tp* means true positive, that is, how often it is relevant, and where the model is correctly identified as relevant. The false positive rate *fp* means the number of unrelated results, but the model incorrectly marks them as relevant. Similarly, the negative error rate *fn* indicates how often it is relevant and when the model is incorrectly marked as inappropriate. The precision and recall is also calculated for ULBERT, which are presented next.

#### 4.4.3 F-score

The F-score, also known as the F1-score, is a metric of how accurate the model is in each data set. The F-score is a popular benchmark for evaluating information retrieval systems such as IR systems and machine learning models, especially in natural language processing. The ideal model has an F-score of 1. The formula for the standard F1 score is the harmonic average of accuracy and completeness given in Equation 4.6. The F-Score is calculated for our model according to this equation.


(8) $$\begin{array}{l} F-score=\frac{2}{\frac{1}{recall}*\frac{1}{precision}}\\ \;=2*\frac{precision*recall}{precision+recall}\\ \;=\frac{tp}{tp+1/2(fp+fn}. \end{array}$$

Our focus is on these metrics as these directly assess the system's ability to retrieve relevant documents and avoid irrelevant ones, reflecting the binary relevance scenario common in constitutional interpretation. While metrics like Mean Reciprocal Rank (MRR) and Normalized Discounted Cumulative Gain (NDCG) (Wang et al., 2023) are valuable for graded relevance. The NDCG metric calculates the total quality of the ranked list or relevancy, while the MRR focuses only on the first relevant result. The NDCG gives us the overall quality of relevant responses retrieved through our model.

#### 4.4.4 Normalized discounted cumulative gain

Normalized Discounted Cumulative Gain (NDCG) is a metric that evaluates the quality of responses of an IR system (Wang et al., 2023). The formula for this metric is given in Equation 9, which is the ratio of Discounted Cumulative Gain (DCG) and Ideal Discounted Cumulative Gain (IDCG). Suppose we have a ranked list of documents assigned with some relevant score. In that case, DCG is calculated using the following equation, where *RelScore*_*k*_ is the appropriate score at position k and log_2_(*k*+1) is the discount factor in decreasing the impact of low-ranked documents.


(9) $$\begin{array}{l} NormalDiscCumuGain\left(NDCG\right)=\frac{DiscCumuGain\left(DCG\right)}{IdealDiscCumuGain\left(IDCG\right)} \end{array}$$

The IDCG has the same equation as the DCG in Equation 10, with the difference that it considers the documents in descending order, which means it assumes all the relevant results in optimal order from higher rank to lower rank. The range of the NDCG is between 0 and 1, where 1 means a highly relevant document and 0 means not relevant.


(10) $$\begin{array}{l} DCG=\overset{N}{\underset{k=1}{\sum }}\frac{\left(RelScore_{k}\right)}{\log_{2}\left(k+1\right)}. \end{array}$$

For our model, the documents are ranked by the cosine similarity value discussed in Section 4.4.1 and Example 6.1. This ranking seems an Ideal Relevant Score (IRS) for the NDCG. The top 5 queries from Table 3 are tested to evaluate NDCG (see Appendix 6.3). A scale from 0 to 3 is given below, which is used to take the opinion of domain experts on IRS-based retrieved documents of our model. This opinion is recorded as a Relevant Score (RS). This Relevant Score is taken explicitly for the selected five queries against the documents returned by our model. This Relevant Score is taken through experts in our law community. They marked the RS score according to the following scale.

- Top 1: Score = 3 (High relevancy).
- Top 2 to 3: Score = 2 (Average relevancy).
- Top 4 to 5: Score = 1 (Low relevancy).
- Top 6 to below: Score = 0 (No relevancy).

### 4.5 Results and discussion

ULBERT model is trained on the acquired data set (Table 2). This model uses the stack of encoders known as transformers, excluding decoders, as decoders are ignored in BERT. Each encoder incorporates two layers inside the attention layer and a position-wise feedforward layer. We developed and ran ULBERT with the base version of BERT using 12 stacks. For testing, we put 100 queries (Table 3) separately to check the results of our model. We use Google Cloud Platform (GCP) (Mok et al., 2021) account and Google Cloud Storage (GCS) (Mondal et al., 2021) bucket for persistent storage of training data and models. The source code of the research project is written using Python and Bash commands to run in the ColabJupyter environment. The complete architecture of ULBERT is shown in Figure 1 in Section 3. The maximum query length is set to 256 tokens with a “Reduce Mean” pooling strategy and the dimension of hidden layers to 768. The configuration of the experiment environment was as follows. The Windows 10 Professional Edition is used with GPU as GCP & GCS & Colab Jupyter, 8GB memory, Python 3.6, and TensorFlow 1.15.0. The input pipeline configurations include TRAIN_BATCH_SIZE = 128, MAX_PREDICTIONS = 20, MAX_SEQ_LENGTH = 128, and MASKED_LM_PROB = 0.15. The training procedure configurations are VAL_BATCH_SIZE = 64, LEARNING_RATE = 2*e*^−5^, TRAIN_STEPS = 1,000,000, SAVE_CHECKPOINTS_STEPS = 2,500, and NUM_TPU_CORES = 8. The process of the experiment is explained in Section 3.6. The experiment results under Nearest-Neighbor Retrieval or Euclidean Distance are given in Table 4 as follows.

**Table 4:** Results under nearest neighbor retrieval or Euclidean distance.

**Threshold**	**Recall**	**Precision**	**Similarity**
θ = 0.2	81%	95%	0.8541
θ = 0.1	85%	92%	0.8836

**Figure 11:**
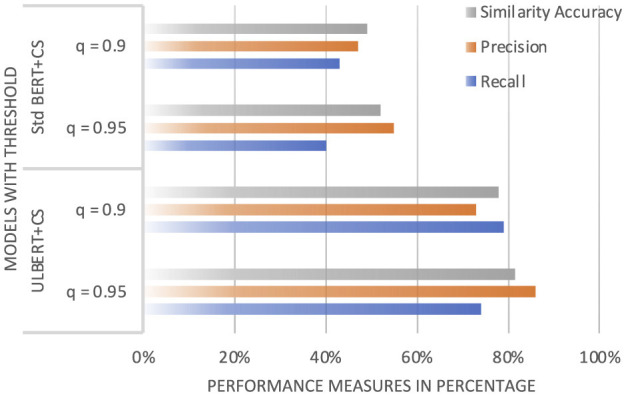
Performance Evaluation: Standard BERT vs. ULBERT.

To evaluate the system performance, this work examined 100 queries from the test data (see Table 3) to test the accuracy and speed of the system memory. Different thresholds were used in the experiment and observed higher equivalence on low thresholds when calculating equivalence according to the Euclidean distance. When calculating the product content (cosine parity), our approach achieved higher parity for a higher threshold, as shown in Table 5. Further discussion of the higher threshold is presented in Section 4.5.1 and Figure 11.

**Table 5:** Results under inner product or cosine similarity.

**Threshold**	**Recall**	**Precision**	**Similarity**
θ = 0.95	74%	86%	0.8144
θ = 0.90	79%	73%	0.7790

**Figure 12:**
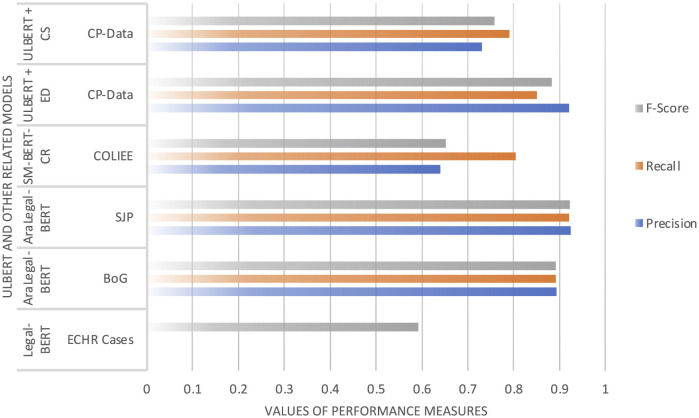
Comparison of ULBERT model with other models.

In the experiments, recall, precision, and accuracy shifted noticeably with changes to the threshold. As shown in Table 4, setting the threshold to 0.1 led to a boost in accuracy but a drop in precision. Table 5 revealed almost the opposite pattern, raising the similarity threshold improved accuracy but caused recall to fall. From a recall standpoint, the Euclidean distance measure consistently delivered higher and more stable results, while keeping accuracy within an acceptable range. Taken together, these findings suggest that using Euclidean distance for similarity calculation works better for this system than the Cosine Similarity metric. With a fixed threshold of 0.1, the approach achieved 85% recall, 92% precision, and 88% similarity accuracy. General discussion with respect to Euclidean Distance and Cosine Similarity of the ULBERT model with other models is done in Section 4.5.2 and also depicted in Figure 12.

**Figure 13:**
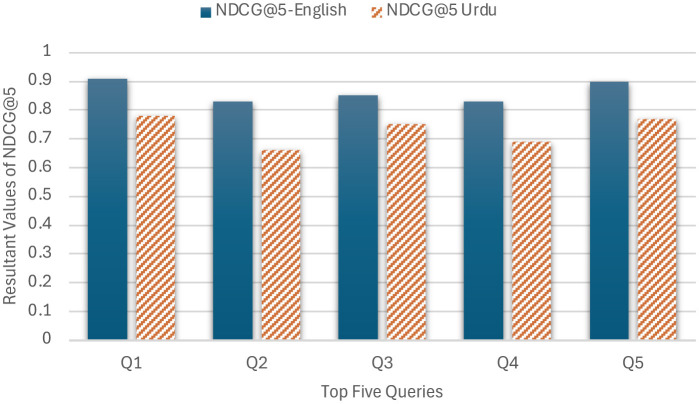
Performance of English and Urdu Queries on ULBERT.

The averages of the NDCG@5 for the English and Urdu queries are 0.86 and 0.73, respectively, as shown in Table 6. This shows that the model's ranking for English documents is better than that of Urdu documents. Moreover, Queries Q2 and Q4 underperformed the other queries, especially for Urdu, as shown in Figure 13. One understandable reason is the data size in the pre-trained base-BERT model, which is fine-tuned. This only has data for English from BooksCorpus and Wikipedia, and there is no such data for Urdu in our experiment, except for our dataset. Urdu queries have higher linguistic variability, which can cause inconsistent document rankings. As the Urdu language is more inflectional than English, the tokenizer of our model can over-fragment the tokens, which can lead to low-quality vectors and affect the ranking of documents. Another reason is better vocabulary, better embeddings, and lower vocabulary, poor embeddings, because the model has to rely on subwords or unknowns. Urdu queries are usually longer and can contain dynamic semantics due to idiomatic expressions, and ranking cannot be assured due to improper embeddings. This can be improved by expanding the dataset. We believe that stronger results in Urdu can be achieved with proper preprocessing. Performance could also be boosted by fine-tuning hyperparameters, as successfully done for Cosine Similarity and Euclidean Distance in Appendix Table 8 and Table 5. A direct comparison of NDCG@5 scores with the models in Table 7 isn't possible, since those models were mainly evaluated using the F1-Score. However, the authors of Legal-BERT (available only in English) (Chalkidis et al., 2020) did share NDCG@5 results, which are reproduced and compared in Figure 11. While the model here shows stronger ranking performance than Legal-BERT, it still falls short in Urdu—a gap largely due to the domain-specific advantage built into Legal-BERT.

**Table 6:** Results of NDCG@5 top five English and Urdu queries from Table 3.

**Query**	**NDCG@5-English**	**NDCG@5 Urdu**
Q1	0.91	0.78
Q2	0.83	0.66
Q3	0.85	0.75
Q4	0.83	0.69
Q5	0.90	0.77
**Average**	0.86	0.73

**Table 7:** Results of the ULBERT using Euclidean distance (ED) and cosine similarity (CS).

**Model**	**Corpus**	**No. of docs**	**Size**	**Precision**	**Recall**	**F1**	**Mod-type**
Legal-BERT	ECHR Cases	12554	0.5GB	–	–	0.592	Retrieval
AraLegal-BERT (Al-qurishi et al., 2022)	BoG	–	350KB	0.89276	0.89173	0.89098	Classifier
AraLegal-BERT (Al-qurishi et al., 2022)	SJP	–	350KB	0.92395	0.92133	0.92210	Classifier
SM-BERT-CR (Vuong et al., 2023)	COLIEE	181	–	0.6395	0.8050	0.6528	Retrieval
ULBERT + ED	CP-Data	600 pages	13.66MB	0.92	0.85	0.8836	Retrieval
ULBERT + CS	CP-Data	600 pages	13.66MB	0.73	0.79	0.7588	Retrieval

#### 4.5.1 Standard BERT with the ULBERT dataset

The ULBERT has a vocabulary that is different from the standard BERT benchmark, as it was trained on Wikipedia and the book corpus in 2018. However, in our test data, the two models are evaluated (the standard BERT with its own trained embeddings named Std-BERT and the standard BERT with the embeddings of our model named ULBERT). The results are plotted in Figure 13. On the 100 queries given of the English test data, the standard BERT model achieved partial accuracy with 40% recall, 55% precision, and 51% accuracy using cosine similarity. This is due to the different training data of standard BERT and ULBERT. On the other hand, ULBERT performed better on test data with 79%, 73%, and 77% of recall, precision, and accuracy (Figure 13) due to the existence of embeddings related to the legal dataset.

#### 4.5.2 The ULBERT and others

Tools like the AILawyer mobile app (see Section 1) show clear limitations. Its claim to cover “150 constitutions” is hard to confirm because the app does not reveal its data sources. As a commercial product, access is limited to paying users, and it has not been validated in peer-reviewed research; its technical details appear only in proprietary blog posts. This gap underscores the need for open, transparent, and academically grounded tools, especially for underserved areas. To the authors' knowledge, no legal IR system exists for the Constitution of Pakistan, so a direct comparison with AILawyer was not possible. A comparative study with related work is presented in Section 2. The Legal-BERT (Chalkidis et al., 2020) uses the batch size of 4,8,16, 32, an unfixed number of epochs, the learning rate of 1*e*^−5^ other than the standards from 2*e*^−5^ to 5*e*^−5^ to remove the local minimum, and a dropout rate of 0.2 for regularization. With these configurations in the standard BERT and the data set of ECHR cases, it achieves a 59.2% F1-score. Precision and recall are not reported (Chalkidis et al., 2020). When considering recall more stable than precision, the results are displayed in Table 7, which shows that ULBERT outperforms Legal-BERT with 29% of F-Score. The difference is due to the corpus's configuration settings, nature, and size. ULBERT settings are the state-of-the-art given in Section 4, and the size of the data set is also tiny, but the size does not matter much to the BERT model. Although the Legal-BERT has the standard settings, the corpus size is also significant, which might not be appropriately addressed with the standard settings. The common attribute between Legal-BERT and our ULBERT working model is the model type. Both are document retrieval models in contrast to the AraLegal-BERT (Al-qurishi et al., 2022) discussed next.

**Figure 14:**
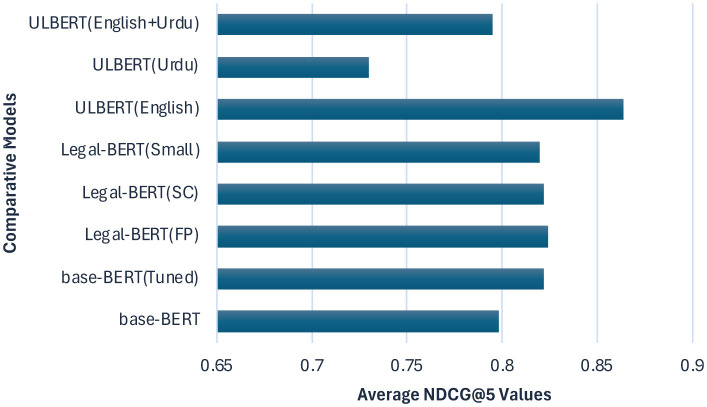
Models Comparison Over Average NDCG@5.

The configuration settings for the AraLegal-BERT (Al-qurishi et al., 2022) include 500K steps, vocabulary size 64K, 50 epochs, 8 GPUs, batch size 512, learning rate 1e-1 to 5*e*^−5^, 12, and 24 layers, etc. The data set size was initially reported as 4.5GB, then filtered, and the final size of the training set was reported in terms of 13.7 million sentences, not in GBs. AraLegal-BERT, a document classifier and not a document retrieval model, has precision, recall, and F-score for the BoG dataset as 0.89276, 0.89173, and 0.89098, respectively. Similarly, the SJP dataset has a precision of 0.92395, a recall of 0.92133, and an F-score of 0.92210. This AraLegal-BERT has quite outperforming results, but the reason for the same precision, recall, and f-score needs to be discussed. It means that the number of false positives and false negatives is the same, which is rare. Regarding cosine similarity, AraLegal-BERT performed much better than our ULBERT model with almost 18% precision, 11% recall, and 15% F-score after subtracting from the average. Similarly, ULBERT has performed better in Euclidean distance, with only nearly 1.16% precision. The other model has the advantage of 5.65% recall and 2.29% accuracy. We have presented comparative statistics only due to the legal domain, as seen in Table 7 and Figure 14.

Technically, the design of the SM-BERT-CR (Vuong et al., 2023) is different from our model ULBERT, except for using the BERT model. The dataset (COLIEE) contained the Federal Court of Canada cases. Despite fine-tuning the pre-trained BERT (Devlin et al., 2018), the TextRank algorithm is used to extract decision-sentence, and to obtain its embedded vectors, GLOVE (Pennington et al., 2014) with 300 dimensions is used. Hence, technically, this model is different from the models proposed in Chalkidis et al. (2020), Al-qurishi et al. (2022), and Masala et al. (2021). However, the precision, recall, and F-score on the COLIEE 2020 test set are 0.6395, 0.8050, and 0.6528, respectively. Our ULBERT model has almost the same recall as the SM-BERT-CR model when the measure is cosine similarity, but outperforms the model for precision and F-score with 9.1% and 10.6%, respectively. In the case of our advocated Euclidean distance measure, ULBERT outperformed the SM-BERT-CR model by 28.1%, 4.5%, and 23.1% precision, recall, and F-score, respectively.

Our legal document-based IR system, ULBERT, leverages the power of Bidirectional Encoder Representations from Transformers (BERT) to enhance information retrieval in the legal domain. We develop a model that performs on par with AraLegal-BERT while surpassing the capabilities of Legal-BERT and SM-BERT-CR.

#### 4.5.3 Scalability and computational considerations

**Scalability of the vector-based approach:** ULBERT's vector-based design offers inherent scalability advantages over traditional keyword-based IR systems. The offline computation of document embeddings allows for rapid online query processing, as only the query vector needs to be compared against the pre-built index. This approach is particularly beneficial for complex or lengthy queries, where keyword matching would be significantly slower.

**Computational resources and latency:** While a detailed benchmark analysis on huge datasets was outside the scope of this initial study, our internal testing indicates that ULBERT achieves sub-second response times for typical queries on the Constitution of Pakistan dataset, using standard server hardware. This demonstrates satisfactory performance for legal professionals using interactively.

**Figure 15:**
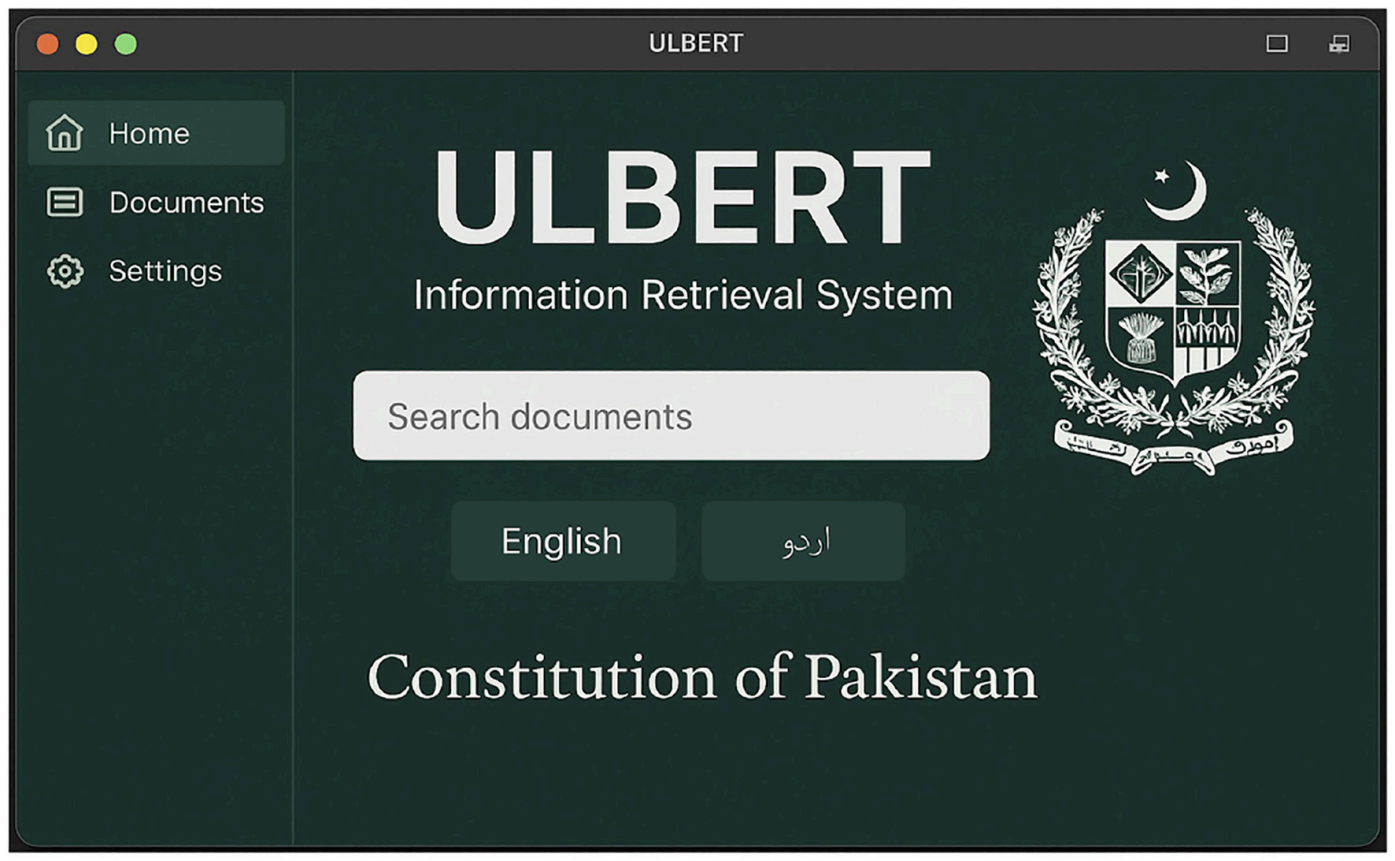
ULBERT's Dashboard.

**Offline processing:** The creation of document embeddings in ULBERT is performed offline. This design choice, which separates indexing from query processing, improves online latency. The screenshot of the dashboard for an end user is displayed in the Figure 15.

#### 4.5.4 Bias and fairness considerations

We used the SKlean logistic regression, which has the value of *class*_*weight* = *dict*, our vocabulary extracted by our model. This vocabulary contains tokens along with the document IDs. When we set *class*_*weight* = *dict*, it calculates the total number of documents (*n*_*classes*) and tokens related to each document (*n*_*samples*). It uses this formula *n*_*samples*/(*n*_classes*np.*bincount*(*y*)) to adjust the dataset's class imbalances by default. This part *np*.*bincount*(*y*) returns the frequency of occurrences of each class in the target variable y. Through this formula, we are assigning higher weights to the minority class and lower weights to the majority class, which helps to improve the performance of the model on the classes.

#### 4.5.5 Ablation and augmentation study

This section briefly describe the BERT components, which are either completely removed (Ablation) or added (Augmentation) for developing the proposed ULBERT model.

- For information retrieval, the decoder of the BERT is also in use and producing promising results like T5 (Raffel et al., 2020) and others. Still, completely ignoring the BERT decoder in this early stage of our development for an under-resourced language, Urdu. While having our domain-specific dataset, this practice is recommended by the designers of the BERT and the later researchers (Devlin et al., 2018; Chalkidis et al., 2020). It lowers the computation time of the model during training.
- The WordPiece tokenizer used in BERT has trouble with out-of-vocabulary words, over-splitting or odd lemmatization, handling complex or domain-specific terms, and maintaining punctuation and cross-language consistency. These issues match prior reports (e.g., Qarah and Alsanoosy, 2024; Deepa, 2021) and the authors' own experience, especially for particular domains and multilingual cases. For that reason, the authors removed WordPiece from their options and adopted the SentencePiece tokenizer (available on GitHub^13^), which is language-independent and better suited to their needs.
- For downstream tasks like ours, it is recommended to overcome issues like the complexity of the classification layer, reduce overfitting, get efficient fine-tuning, multi-task learning, etc. These benefits are achieved by breaking the connection between the feed-forward neural network and softmax function. We introduced a new layer with logistic regression (See Figure 9), which gives us the benefits of simplicity of the classification layers, reducing overfitting, and efficient fine-tuning.
- Rather than relying on BERT's usual embedding layer to index documents alongside tokens and sentences, we introduce a dedicated fourth embedding that complements the model's three standard embeddings. This extra layer handles document-level indexing and works together with the token, segment, and position embeddings. See Figure 8 in Section 3.6 for a visual.

#### 4.5.6 Limitations and future work

**Scope of evaluation:** We acknowledge that the current evaluation is limited to a single dataset, the Constitution of Pakistan. While this document represents a complete and complex legal framework, testing ULBERT on other Pakistani legal texts (e.g., statutes, case law) is crucial for future work. This would assess the model's generalizability across different legal domains and document structures within the Pakistani legal system.

**Comparison with other systems:** Direct comparison with systems like Legal-BERT and LexNLP is complicated by the fundamental differences in language (Urdu vs. English) and legal systems (Pakistani vs. U.S./European). Future research could explore cross-lingual and cross-jurisdictional evaluation methods, but this presents significant methodological challenges.

**Resource availability:** Developing such an IR system for low-resourced languages like Urdu requires significant effort. ULBERT has laid the foundation stone for future research.

**Expansion of datasets:** Acquiring, cleaning, and pre-processing additional Urdu legal datasets is a resource-intensive task. Future work will expand the dataset to include a wider range of legal documents, enhancing the system's coverage and robustness.

**Alternative architectures:** While our fine-tuning approach yielded substantial results, exploring other model architectures and training strategies could further improve performance. This could include investigating other transformer-based models or incorporating domain-specific knowledge into the model.

**User-centric evaluation:** A key direction for future work is to conduct a thorough user-centric evaluation of ULBERT. While the current study establishes strong technical performance, assessing usability, relevance from a legal professional's perspective, and overall user satisfaction is crucial. We plan to conduct user studies involving legal professionals (lawyers, law students, researchers) performing realistic legal research tasks. These studies will incorporate task completion success metrics, qualitative feedback through questionnaires and interviews, and potentially a comparative analysis with existing legal research methods. This will provide valuable insights into ULBERT's practical utility and inform further development.

**Handling ambiguity in Urdu legal queries:** Urdu legal text, particularly within the Constitution of Pakistan, presents challenges in handling ambiguous queries. For instance, "صدر" (Sadar) can mean both “President” and “Cantonment” but with different meanings. Articles using these words are present in the Constitution. The ULBERT's current design partially addresses this via its vector embeddings and attention mechanism. However, limitations remain, especially with complex syntax (e.g., queries about the impeachment process under Article 47) or mixed Urdu-English queries. Sentences with long dependencies and multi-clauses are difficult to understand semantically, even for BERT. For example:

Urdu Sentence: ضمیمہ میں نقل کردہ قرار داد مقاصد میں بیان کردہ اصول اور احکام کو بذریعہ ہذا دستور کا مستقل حصہ قرار دیا جاتا
English Translation: The principles and the orders stated in the resolution copied in the appendix are declared to be the permanent part of the constitution by this rule.

First, it is a passive sentence; the case is also dative due to the case marker کو “ko.” The question is, what things are declared permanent? The answer is the subject of the sentence, and the BERT is facing difficulty in finding the subject “principles and orders” here. The BERT gives “resolution” as the subject, which is wrong. The issue can be solved easily through tokenizers if the proper tokenizer for Urdu is used. Because Urdu is a case-marked language like German, and by looking at the case marker کو “ko,” the subject can be easily identified.

Future work will focus on enhanced word sense disambiguation, synonymy, loanwords, robust syntactic parsing, multilingual capabilities, and query suggestions to improve handling such ambiguities.

**Error analysis:** While a comprehensive error analysis was beyond this initial study's scope, internal testing revealed key failure modes specific to the Urdu Constitution. Ambiguous queries, such as using "ووٹ" (vote), which can refer to general voting, a vote of no confidence (Article 95), or committee votes, often lead to overly broad retrievals. Complex syntactic structures, like those found in queries about the conditions for emergency rule under Article 232, can cause misinterpretations due to clause relationships. Near-synonyms with subtle legal distinctions, such as "عدالت" (adalat - court) and "عدلیہ" (adliya - judiciary, as in Article 175), can result in incomplete retrieval. Out-of-vocabulary (OOV) words may be missed, particularly newly coined legal terms (e.g., in cybercrime). Finally, users may lack the specific legal phrasing used in the Constitution, such as Article 13's “Protection against double punishment…” instead of the common term “double jeopardy,” leading to retrieval failures. Future work will address these issues through improved word sense disambiguation, robust syntactic parsing, expansion of the legal vocabulary, incorporation of legal knowledge bases, and user interface enhancements for query refinement.

**Integration with legal workflows:** To maximize ULBERT's practical impact, future work will focus on integrating it with existing legal workflows. This includes potential integrations with online legal databases (statutes, case law), case management systems, government portals, e-courts initiatives, and legal research-sharing platforms. Such integrations would enable seamless access to ULBERT's constitutional search capabilities within the broader context of legal research, case preparation, and judicial proceedings. API development and data interoperability will be key considerations.

**Zero-shot or few-shot learning:** ULBERT builds on a base BERT model and is trained on unlabeled data; it can accommodate both zero-shot and few-shot strategies, either directly or indirectly. For zero-shot use, the pre-trained embeddings from the base BERT are already available; additionally, short prompts or text descriptions could be added as an extra layer in future versions, and ULBERT already includes a mechanism to compare a query with candidate documents. Few-shot learning is not immediately feasible here, since labeled examples for legal Urdu are lacking. A practical path forward is to apply zero-shot methods first to generate pseudo-labels for the unlabeled corpus, then fine-tune ULBERT on those pseudo-labeled examples.

## 5 Conclusion and future directions

ULBERT, a BERT-based information retrieval system tailored for the Constitution of Pakistan, successfully demonstrates the potential of applying modern NLP techniques to Urdu legal documents. This represents a notable advancement in creating accessible IR systems for a previously underserved domain. While acknowledging limitations in directly calculating sentence probabilities with BERT, ULBERT's feature extraction approach, particularly its effective use of Euclidean distance for similarity measurement, provides robust information retrieval, achieving 85% recall and 88% F1-score. This functional system offers a valuable resource for legal professionals, researchers, and citizens, facilitating easier access and understanding of Pakistani constitutional law.

Future work on ULBERT will build on its current strengths while pushing into new territory. The team plans to test the model on a wider range of Pakistani legal texts, making sure it works reliably across the legal landscape. A major priority is tackling the natural ambiguity found in Urdu legal language by using tools like advanced word sense disambiguation, better syntactic parsing, and rich legal knowledge bases. Equally important, continuous feedback from legal professionals will guide improvements, ensuring the system is genuinely useful in practice. Efforts will also go into expanding the vocabulary, finding ways to integrate smoothly with existing legal workflows, and experimenting with new model designs to boost performance.

## Data Availability

Publicly available datasets were analyzed in this study. This data can be found here: https://www.pakistani.org/pakistan/constitution/, https://learningpartnership.org/resource/constitution-pakistan-document-urdu.

## Footnotes

^1^ https://www.courtlistener.com/

^2^ http://www.findlaw.com/

^3^ http://www.justia.com/

^4^ http://www.law.cornell.edu/

^5^ https://www.fastcase.com/

^6^ https://www.aiattorney.com.pk/

^7^ https://ailawyer.pro/

^8^ https://www.pakistani.org/pakistan/constitution/

^9^ https://www.learningpartnership.org/resource/constitution-pakistan-document-urdu

^10^ https://gradient.run/

^11^ https://colab.research.google.com/

^12^ https://www.kaggle.com/

^13^ https://github.com/google/sentencepiece
