# Supplementary material for: ULBERT: a domain-adapted BERT model for bilingual information retrieval from Pakistan's constitution
Source: Front Big Data. 2025 Sep 22;8:1448785. doi: 10.3389/fdata.2025.1448785 (PMC12497596; doi:10.3389/fdata.2025.1448785)
Supplement: Supplementary file 1 [file Data_Sheet_1.pdf]

Supplementary Appendix for:

ULBERT: A Domain-Adapted BERT Model for Bilingual Information Retrieval from Pakistan's Constitution

Qaiser Abbas[1], Waqas Nawaz[2,*], Sadia Niazi[3] and Muhammad Awais[4]

September 10, 2025

Contents

A Simulated Example for IR **3**

B Importance of Cosine Similarity: Identifying Articles Defining Essential Rights **5**

C Example Illustration For Normalized Discounted Cumulative Gain (NDCG) **6**

A Simulated Example for IR

This section highlights simulated embeddings and calculations for demonstration purposes. The BERT model initially uses much higher-dimensional embeddings, e.g., 768 dimensions in embedding layers and much more during attention heads and FFNN.

- Scenario: To build an information retrieval system for the Constitution of Pakistan, in order to find articles relevant to a user's query.
- Tools (Conceptual): Extended BERT Model (ULBERT): Fine-tuned on the Constitution of Pakistan (both Urdu and English versions). This model is crucial for creating meaningful embeddings.
- Embedding Generation Function: A function (conceptually representing the BERT model's output) that takes text as input and returns a vector embedding.
- Cosine Similarity Function: A function calculates the cosine similarity between two vectors.

Example:

1. The Query:

   Urdu: "بنیادی حقوق کی ضمانت"

   English: Guarantee of fundamental rights

2. Relevant Articles (Excerpts - Simplified for Brevity):

   Document 1 (Article 9 - Security of Person):

   Urdu: "کسی شخص کو زندگی یا آزادی سے محروم نہیں کیا جائے گا سوائے قانون کے مطابق۔"
   English: ”No person shall be deprived of life or liberty save in accordance with law.”

   We are mainly focusing on the Urdu part for this demonstration.

   Document 2 (Article 19 - Freedom of Speech):

   Urdu: "ہر شہری کو تقریر اور اظہار رائے کی آزادی کا حق حاصل ہوگا، بشرطیکہ اس سے..."
   English: ”Every citizen shall have the right to freedom of speech and expression, subject to...”

   Document 3 (Article 175 - Establishment and Jurisdiction of Courts):

   Urdu: "پاکستان کے لیے ایک عدالت عظمیٰ ہوگی جسے سپریم کورٹ آف پاکستان کہا جائے گا۔"
   English: ”There shall be a Supreme Court of Pakistan.”

   Again, we are mainly using the Urdu text.

3. Simulated Embeddings (5-Dimensional for Illustration): Let us assume our embedding function (representing our BERT model) produces the following 5-dimensional embeddings:

   Query Embedding (Q): [0.9, 0.7, -0.2, 0.5, 0.1]

   Document 1 Embedding (S_1): [0.8, 0.6, -0.1, 0.4, 0.2]

   Document 2 Embedding (S_2): [0.7, 0.8, 0.1, 0.3, -0.2]

   Document 3 Embedding (S_3): [0.1, 0.2, 0.9, -0.3, 0.4]

4. Cosine Similarity Calculations: We'll use the cosine similarity formula: $\frac{Q.S}{|Q||S|}$

Cosine Similarity (Q, S_1):

$Q.S_1 = (0.9 * 0.8) + (0.7 * 0.6) + (-0.2 * -0.1) + (0.5 * 0.4) + (0.1 * 0.2) = 0.72 + 0.42 + 0.02 + 0.20 + 0.02 = 1.38$

$|Q| = \sqrt{0.9^2 + 0.7^2 + (-0.2)^2 + 0.5^2 + 0.1^2} = \sqrt{0.81 + 0.49 + 0.04 + 0.25 + 0.01} = \sqrt{1.6} = 1.265$

$|S_1| = \sqrt{0.8^2 + 0.6^2 + -0.1^2 + 0.4^2 + 0.2^2} = \sqrt{0.64 + 0.36 + 0.01 + 0.16 + 0.04} = \sqrt{1.21} = 1.1$

Cosine Similarity $(Q, S_1) = 1.38/(1.265 * 1.1) = 0.991$

Cosine Similarity (Q, S_2):

$Q.S_2 = (0.9 * 0.7) + (0.7 * 0.8) + (-0.2 * 0.1) + (0.5 * 0.3) + (0.1 * -0.2) = 0.63 + 0.56 - 0.02 + 0.15 - 0.02 = 1.3$

$|Q| = 1.265$ (same as before)

$|S_2| = \sqrt{0.7^2 + 0.8^2 + 0.1^2 + 0.3^2 + -0.2^2} = \sqrt{0.49 + 0.64 + 0.01 + 0.09 + 0.04} = \sqrt{1.27} = 1.127$

Cosine Similarity $(Q, S_2) = 1.3/(1.265 * 1.127) = 0.913$

Cosine Similarity (Q, S_3):

$Q.S_3 = (0.9 * 0.1) + (0.7 * 0.2) + (-0.2 * 0.9) + (0.5 * -0.3) + (0.1 * 0.4) = 0.09 + 0.14 - 0.18 - 0.15 + 0.04 = -0.06$

$|Q| = 1.265$(same as before)

$|S_3| = \sqrt{0.1^2 + 0.2^2 + 0.9^2 + -0.3^2 + 0.4^2} = \sqrt{0.01 + 0.04 + 0.81 + 0.09 + 0.16} = \sqrt{1.11} = 1.054$

Cosine Similarity $(Q, S_3) = -0.06/(1.265 * 1.054) = -0.045$

5. Results and Interpretation:

   Query vs. Document 1 (Article 9): Cosine Similarity 0.991 (Very High)

   Query vs. Document 2 (Article 19): Cosine Similarity 0.913 (High)

   Query vs. Document 3 (Article 175): Cosine Similarity -0.045 (Very Low - Essentially Unrelated)

6. Analysis:

   - High Similarity (Document 1): The system correctly identifies Article 9 ("Security of Person," which relates directly to fundamental rights) as highly relevant. The embeddings capture the semantic overlap between "بنیادی حقوق کی ضمانت"(guarantee of fundamental rights) and "کسی شخص کو زندگی یا آزادی سے محروم نہیں کیا جائے گا"(no person shall be deprived of life or liberty). Even though the exact words aren't identical, the underlying meaning is very close.
   - High Similarity (Document 2): Article 19 (Freedom of Speech) is also correctly identified as relevant, although slightly less than Article 9. Freedom of speech is fundamental, so the semantic link is strong.
   - Low Similarity (Document 3): Article 175 (Establishment of Courts) is correctly identified as irrelevant. The topic of court structure is semantically distant from the concept of fundamental rights.

7. Why This Works (Recap with Urdu Context)?:

   - The model ranks the documents against any query based on the cosine similarity score, and then the top-ranked documents are delivered as the response.

- Semantic Matching: BERT captures the meaning of the Urdu phrases, not just the surface-level words. This is how it connects "بنیادی حقوق" (fundamental rights) with concepts like "زندگی یا آزادی" (life or liberty) and "تقریر اور اظہار رائے کی آزادی" (freedom of speech and expression).
- Contextual Understanding: BERT understands that "حقوق" (rights) in the query refers to fundamental rights within the constitutional context.
- Urdu Grammar and Word Order: The differences in sentence structure between the query and the articles are handled effectively by BERT's contextual understanding.

This example, though using simulated embeddings, demonstrates the principle of using cosine similarity with BERT embeddings for information retrieval in Urdu legal documents. We also calculate Euclidean Distance, which is discussed in the main article. The calculations show how documents with semantically similar content to the query receive high cosine similarity scores, while unrelated documents receive low scores. This approach effectively addresses the challenges of Urdu's linguistic features and provides a robust way to find relevant information within the Constitution of Pakistan. A real-world system with an appropriately trained BERT model would perform even more accurately, as observed in the empirical evaluation section of the ULBERT.

B Importance of Cosine Similarity: Identifying Articles Defining Essential Rights

We present a scenario using the Constitution of Pakistan to further validate our system's robustness in the context of cosine similarity. Here, the user asks, "ضروری حقوق کیا ہیں؟" (meaning "What are essential/necessary rights?"). This query, while related to fundamental rights, uses different terminology.

Our system, employing the same extended BERT model, processes this new query and compares it to the constitutional articles:

- Article 9 (Security of Person): "کسی شخص کو زندگی یا آزادی سے محروم نہیں کیا جائے گا..." (Protecting life and liberty).
- Article 19 (Freedom of Speech): "ہر شہری کو تقریر اور اظہار رائے کی آزادی..." (Guaranteeing freedom of speech).
- Article 175 (Establishment of Courts): "پاکستان کے لیے ایک عدالت عظمیٰ ہوگی..." (Establishing the Supreme Court).

Using cosine similarity to compare the semantic embeddings generated by our model, we obtain the following results, shown in Table 1.

Table 1: Cosine similarity results for a given query.

Article	Cosine Similarity with Query	Interpretation
Article 9 (Security of Person)	0.985	Very High Relevance
Article 19 (Freedom of Speech)	0.898	High Relevance
Article 175 (Establishment of Courts)	-0.038	Very Low/No Relevance

The results demonstrate the system's resilience to variations in user phrasing. Despite the shift from "guarantee of fundamental rights" to "what are essential rights," the system correctly identifies Articles 9 and 19 as highly relevant. The core semantic connection – essential or

fundamental rights – is accurately captured and reflected in the high cosine similarity scores. Article 175, concerning court structure, remains correctly classified as irrelevant.

This example highlights the system's ability to understand the underlying intent of the user's query, even when expressed using different words. This robustness is crucial for a practical information retrieval system, where users may not always use the precise legal terminology found in the documents. Combining BERT's contextual understanding and cosine similarity's semantic matching ensures accurate and reliable retrieval, even with nuanced queries in Urdu. This further strengthens the case for the effectiveness of our approach in accessing legal information within the Constitution of Pakistan.

C Example Illustration For Normalized Discounted Cumulative Gain (NDCG)

This section illustrates the evaluation of the selected five queries of English and Urdu from the Queries Test Data table, see the main document for further discussion on it.

Table 2: Selected Queries for the NDCG

Q No.	Question	سوال
1	What are the essential rights?	بنیادی حقوق کیا ہیں؟
2	The composition of the Parliament of Pakistan under the charter of the Islamic Republic of Pakistan.	اسلامی جمہوریہ پاکستان کے آئین کے تحت پارلیمنٹ آف پاکستان کی تشکیل۔
3	What are the safeguards for residents regarding Preventive Detention?	احتیاطی حراست کے حوالے سے شہریوں کے لیے کیا حفاظتی اقدامات ہیں؟
4	The powers and role of the president of Pakistan under the constitution of the Islamic Republic of Pakistan, 1993, 1994, and 1997.	آئین اسلامی جمہوریہ پاکستان 3991،4991،7991’ کے تحت صدر پاکستان کے اختیارات اور کردار۔
5	The powers and jurisdiction of the High Courts under Article 199 of the Constitution of the Islamic Republic of Pakistan.	اسلامی جمہوریہ پاکستان کے آئین کے آرٹیکل 991 کے تحت ہائی کورٹس کے اختیارات اور دائرہ اختیار۔

A ranked list of documents is retrieved from our model in descending order of cosine similarity (CS), which is known as IRS. Similarly, the score received from the experts is RS. The IRS of the model is divided into ranges to align with the RS, as follows:

- Score =3 (IRS from 0.70 to 1.00)
- Score =2 (IRS from 0.40 to 0.69)
- Score =1 (IRS from 0.00 to 0.39)
- Score =0 (IRS less than 0.00)

Q1: What are the essential rights?

Relevance Score (RS): [3,1,1,0,0]

DCG=$\frac{3}{log_2(1+1)}+\frac{1}{log_2(2+1)}+\frac{1}{log_2(3+1)}+\frac{0}{log_2(4+1)}+\frac{0}{log_2(5+1)} = 3+0.63+0.5+0+0 = 4.13$

Ideal Relevant Score (IRS): [3,1,1,1,0]

Table 3: English Queries from Table 2

Query No.	Top 5 Model Predictions	IRS Rank	RS Rank
Q1	D4,D3,D1,D5,D38	[3,1,1,1,0]	[3,1,1,0,0]
Q2	D8,D9,D7,D12,D33	[3,1,1,1,1]	[3,1,1,0,0]
Q3	D4,D2,D17,D31,D32	[3,1,1,0,0]	[3,0,1,0,0]
Q4	D7,D9,D11,D14,D50	[3,2,2,1,1]	[3,1,2,1,0]
Q5	D25,D23,D24,D26,D31	[3,2,1,1,0]	[3,2,0,1,0]

IDCG=$\frac{3}{log_2(1+1)}+\frac{1}{log_2(2+1)}+\frac{1}{log_2(3+1)}+\frac{1}{log_2(4+1)}+\frac{0}{log_2(5+1)} = 3+0.63+0.5+0.43+0 = 4.56$

NDCG=$\frac{4.13}{4.56} = 0.91$

Q2: The composition of the Parliament of Pakistan under the charter of the Islamic Republic of Pakistan.

Relevance Score (RS): [3,1,1,0,0]

DCG=$\frac{3}{log_2(1+1)}+\frac{1}{log_2(2+1)}+\frac{1}{log_2(3+1)}+\frac{0}{log_2(4+1)}+\frac{0}{log_2(5+1)} = 3+0.63+0.5+0+0 = 4.13$

Ideal Relevant Score (IRS): [3,1,1,1,1]

IDCG=$\frac{3}{log_2(1+1)}+\frac{1}{log_2(2+1)}+\frac{1}{log_2(3+1)}+\frac{1}{log_2(4+1)}+\frac{1}{log_2(5+1)} = 3+0.63+0.5+0.43+0.39 = 4.95$

NDCG=$\frac{4.13}{4.95} = 0.83$

Q3: What are the safeguards for residents regarding Preventive Detention?

Relevance Score (RS): [3,0,1,0,0]

DCG=$\frac{3}{log_2(1+1)}+\frac{0}{log_2(2+1)}+\frac{1}{log_2(3+1)}+\frac{0}{log_2(4+1)}+\frac{0}{log_2(5+1)} = 3+0+0.5+0+0 = 3.50$

Ideal Relevant Score (IRS): [3,1,1,0,0]

IDCG=$\frac{3}{log_2(1+1)}+\frac{1}{log_2(2+1)}+\frac{1}{log_2(3+1)}+\frac{0}{log_2(4+1)}+\frac{0}{log_2(5+1)} = 3+0.63+0.5+0+0 = 4.13$

NDCG=$\frac{3.50}{4.13} = 0.85$

Q4: The powers and role of the president of Pakistan under the constitution of the Islamic Republic of Pakistan, 1993. 1994, 1997.

Relevance Score (RS): [3,1,2,1,0]

DCG=$\frac{3}{log_2(1+1)}+\frac{1}{log_2(2+1)}+\frac{2}{log_2(3+1)}+\frac{1}{log_2(4+1)}+\frac{0}{log_2(5+1)} = 3+0.63+1+0.43+0 = 5.06$

Ideal Relevant Score (IRS): [3,2,2,1,1]

IDCG=$\frac{3}{log_2(1+1)}+\frac{2}{log_2(2+1)}+\frac{2}{log_2(3+1)}+\frac{1}{log_2(4+1)}+\frac{1}{log_2(5+1)} = 3+1.26+1+0.43+0.39 = 6.08$

NDCG=$\frac{5.06}{6.08} = 0.83$

Q5: The powers and jurisdiction of the High Courts under Article 199 of the Constitution of the Islamic Republic of Pakistan.

Relevance Score (RS): [3,2,0,1,0]

DCG=$\frac{3}{log_2(1+1)}+\frac{2}{log_2(2+1)}+\frac{0}{log_2(3+1)}+\frac{1}{log_2(4+1)}+\frac{0}{log_2(5+1)} = 3+1.26+0+0.43+0 = 4.69$

Ideal Relevant Score (IRS): [3,2,1,1,0]

IDCG=$\frac{3}{log_2(1+1)}+\frac{2}{log_2(2+1)}+\frac{1}{log_2(3+1)}+\frac{1}{log_2(4+1)}+\frac{0}{log_2(5+1)} = 3+1.26+0.5+0.43+0 = 5.19$

Table 4: Urdu Queries from Table 2

Query No.	Top 5 Model Prediction	IRS Ranking	RS Ranking
Q1	D4,D3,D38,D50,D24	[3,2,1,1,0]	[3,1,0,0,1]
Q2	D8,D7,D6,D9,D50	[3,1,1,1,0]	[3,0,0,0,0]
Q3	D4,D18,D20,D21,D25	[3,2,1,0,0]	[2,0,0,1,3]
Q4	D7,D47,D50,D9,D11	[3,1,0,0,0]	[2,0,1,0,0]
Q5	D25,D24,D26,D27,D22	[3,1,1,1,0]	[3,0,1,0,0]

NDCG=$\frac{4.69}{5.19} = 0.90$

Q1: بنیادی حقوق کیا ہیں؟

Relevance Score (RS): [3,1,0,0,1]

DCG=$\frac{3}{log_2(1+1)}+\frac{1}{log_2(2+1)}+\frac{0}{log_2(3+1)}+\frac{0}{log_2(4+1)}+\frac{1}{log_2(5+1)} = 3+0.63+0+0+0.39 = 4.02$

Ideal Relevant Score (IRS): [3,2,1,1,0]

IDCG=$\frac{3}{log_2(1+1)}+\frac{2}{log_2(2+1)}+\frac{1}{log_2(3+1)}+\frac{1}{log_2(4+1)}+\frac{0}{log_2(5+1)} = 3+1.26+0.5+0.43+0 = 5.19$

NDCG=$\frac{4.02}{5.19} = 0.78$

Q2: اسلامی جمہوریہ پاکستان کے آئین کے تحت پارلیمنٹ آف پاکستان کی تشکیل۔

Relevance Score (RS): [3,0,0,0,0]

DCG=$\frac{3}{log_2(1+1)}+\frac{0}{log_2(2+1)}+\frac{0}{log_2(3+1)}+\frac{0}{log_2(4+1)}+\frac{0}{log_2(5+1)} = 3+0+0+0+0 = 3.00$

Ideal Relevant Score (IRS): [3,1,1,1,0]

IDCG=$\frac{3}{log_2(1+1)}+\frac{1}{log_2(2+1)}+\frac{1}{log_2(3+1)}+\frac{1}{log_2(4+1)}+\frac{0}{log_2(5+1)} = 3+0.63+0.5+0.43+0 = 4.56$

NDCG=$\frac{3.00}{4.56} = 0.66$

Q3: احتیاطی حراست کے حوالے سے شہریوں کے لیے کیا حفاظتی اقدامات ہیں؟

Relevance Score (RS): [2,0,0,1,3]

DCG=$\frac{2}{log_2(1+1)}+\frac{0}{log_2(2+1)}+\frac{0}{log_2(3+1)}+\frac{1}{log_2(4+1)}+\frac{3}{log_2(5+1)} = 2+0+0+0.43+1.16 = 3.59$

Ideal Relevant Score (IRS): [3,2,1,0,0]

IDCG=$\frac{3}{log_2(1+1)}+\frac{2}{log_2(2+1)}+\frac{1}{log_2(3+1)}+\frac{0}{log_2(4+1)}+\frac{0}{log_2(5+1)} = 3+1.26+0.5+0+0 = 4.76$

NDCG=$\frac{3.59}{4.76} = 0.75$

Q4: آئین اسلامی جمہوریہ پاکستان 3991،4991،7991 کے تحت صدر پاکستان کے اختیارات اور کردار۔

Relevance Score (RS): [2,0,1,0,0]

DCG=$\frac{2}{log_2(1+1)}+\frac{0}{log_2(2+1)}+\frac{1}{log_2(3+1)}+\frac{0}{log_2(4+1)}+\frac{0}{log_2(5+1)} = 2+0+0.5+0+0 = 2.50$

Ideal Relevant Score (IRS): [3,1,0,0,0]

IDCG=$\frac{3}{log_2(1+1)}+\frac{1}{log_2(2+1)}+\frac{0}{log_2(3+1)}+\frac{0}{log_2(4+1)}+\frac{0}{log_2(5+1)} = 3+0.63+0+0+0 = 3.63$

NDCG=$\frac{2.50}{3.63} = 0.69$

Q5: اسلامی جمہوریہ پاکستان کے آئین کے آرٹیکل 991 کے تحت ہائی کورٹس کے اختیارات اور دائرہ اختیار۔

Relevance Score (RS): [3,0,1,0,0]

DCG=$\frac{3}{log_2(1+1)}+\frac{0}{log_2(2+1)}+\frac{1}{log_2(3+1)}+\frac{0}{log_2(4+1)}+\frac{0}{log_2(5+1)} = 3+0+0.5+0+0 = 3.50$

Ideal Relevant Score (IRS): [3,1,1,1,0]

IDCG=$\frac{3}{log_2(1+1)}+\frac{1}{log_2(2+1)}+\frac{1}{log_2(3+1)}+\frac{1}{log_2(4+1)}+\frac{0}{log_2(5+1)} = 3+0.63+0.5+0.43+0 = 4.56$

NDCG=$\frac{3.50}{4.56} = 0.77$
